# Clinical validation of the terminology subset for newborns with Peripherally Inserted Central Catheter in Intensive Care

**DOI:** 10.1590/1980-220X-REEUSP-2025-0203en

**Published:** 2025-10-03

**Authors:** Nanete Caroline da Costa Prado, Harlon França de Menezes, Romanniny Hévillyn Silva Costa Almino, Isabele Silva dos Santos, Rebecca Stefany da Costa Santos, Sérgio Danillo Santana de Lima Juraci, Dhyanine Morais de Lima Raimundo, Richardson Augusto Rosendo da Silva

**Affiliations:** 1 Universidade Federal do Rio Grande do Norte, Programa de Pós-graduação em Enfermagem, Natal, RN, Brazil.

**Keywords:** Clinical Nursing Research, Validation Study, Catheterization, Central Venous, Standardized Nursing Terminology, Infant, Newborn

## Abstract

**Objective:**

To clinically validate a terminological subset of the International Classification for Nursing Practice (ICNP®) for newborns (NB) with Peripherally Inserted Central Catheter (PICC), in the Neonatal Intensive Care Unit (NICU).

**Method:**

Clinical validation study of an ICNP terminology subset®. The research was conducted through the following steps: application of the Nursing Process in NB admitted to a NICU, development of case studies, and assessment of agreement among experts. For data analysis, the Kappa coefficient of agreement, the Kruskal-Wallis test, and the intraclass correlation coefficient (ICC) were used.

**Results:**

The evaluation of diagnoses/outcomes and interventions indicated an agreement ranging from almost perfect to perfect, with ICC classified as excellent. The application of the Kruskal-Wallis test revealed the absence of statistically significant differences between the evaluations.

**Conclusion:**

The study clinically validated a subset of 53 nursing diagnoses/outcomes and 110 interventions, demonstrating their applicability in the care of neonates with Peripherally Inserted Central Catheters.

## INTRODUCTION

Currently, the integration of technology into healthcare practices has proven to be of paramount importance, as it contributes to higher-quality care and enhances patient safety, particularly in invasive and high-complexity procedures^(1)^. Among these technologies, the Peripherally Inserted Central Catheter (PICC) stands out as a device indicated for patients requiring prolonged treatment or experiencing difficulties with peripheral venous access^(2)^.

The effectiveness of the PICC is well-documented in the administration of medications and parenteral nutrition, positioning it as a safe and efficient alternative. Thus, ensuring appropriate venous access is a critical factor in the success of the patient’s clinical recovery process^(1,2)^.

The PICC is widely recommended for neonates due to its ease of insertion and the inherent challenges of obtaining peripheral venous access in this age group. Its use has become increasingly prevalent in neonatal care, particularly within Neonatal Intensive Care Units (NICU). Key advantages include: the possibility of bedside insertion without requiring patient transport; reduced discomfort for the neonate; lower infection risk compared to other venous access devices; extended duration of use; and decreased stress from repeated punctures^(2)^.

Insertion of the PICC must be performed by nurses who are duly trained, as stipulated by Resolution No. 258/2001 of the Federal Nursing Council (COFEN). The nursing team is also responsible for device maintenance, aiming to minimize potential complications and ensure patient safety^(3)^.

Meticulous care is essential from the moment of PICC insertion, which requires proper use of Personal Protective Equipment (PPE) and strict adherence to aseptic technique, through to maintenance procedures. These include timely replacement of sterile dressings, flushing to prevent catheter occlusion, and rigorous disinfection during handling to reduce infection risk^(1,3)^.

In this context, various complications may lead to the non-elective removal of the catheter, often associated with venous access devices. A randomized clinical trial involving 46 neonates reported complication rates ranging from 30.7% to 62.2%, with the most frequent being infection (14.2%), catheter occlusion (12%), infiltration (4%), and traction (4%). A similar study with 111 neonates found a 51% complication rate, with improper positioning (25.7%), phlebitis (19.3%), and occlusion (3.7%) being the most prevalent^(4)^.

Nurses play a pivotal role in the planning and execution of care, with the Nursing Process (NP) serving as a strategic tool to promote organized, systematic, and effective care delivery^(5,6)^. The NP comprises five interdependent and interconnected stages: assessment, diagnosis, planning, implementation, and evaluation of nursing care^(7)^.

According to Resolution No. 736/2024 of COFEN, the Nursing Process must be aligned with standardized language systems, such as the International Classification for Nursing Practice (ICNP®), to ensure greater consistency, accuracy, and quality in care delivery^(7,8)^.

The ICNP® plays a vital role in documenting nursing actions in clinical practice, promoting standardization, organization, and precision in care records. This standardization facilitates clear and effective communication among healthcare professionals and supports the systematization of care. ICNP® also enables the development of Terminological Subsets, which compile statements of diagnoses/outcomes (ND/NO) and nursing interventions (NI) tailored to specific patient groups, thereby enhancing the personalization and quality of care^(9)^.

Thus, ICNP® may be defined as a specialized communication system that accurately represents nursing practice, with applicability in both local and global contexts. It directly contributes to the effectiveness of the Nursing Process and serves as a structured language for the collection, storage, and analysis of nursing data, thereby strengthening and elevating the profession.

To improve the structure and efficacy of Terminological Subsets, clinical validation is essential to ensure their practical applicability and relevance to care delivery.

Moreover, for a more robust structure and enhanced effectiveness of Terminological Subsets, it is crucial to adopt a theoretical or conceptual model as a foundation for clinical practice. Among the leading frameworks, Betty Neuman’s model stands out, conceptualizing the individual as a dynamic system composed of interconnected elements and acknowledging the simultaneous influence of internal and external factors on the patient’s health status^(7,9)^.

A previous study developed and content-validated an ICNP® Terminological Subset for neonates with PICC, grounded in Betty Neuman’s theoretical model. This subset was created to standardize nursing documentation and improve the quality of care records in NICU^(7)^.

Subsequently, the need for clinical validation was identified to ensure the applicability and reliability of the terminological subset in practice. Clinical validation assesses whether the developed statements accurately reflect the real conditions of neonates with PICC, ensuring that ND/NO and NI are effective in clinical settings. This process enables necessary adjustments, enhancing the precision of the terminology used and promoting more targeted, safe, and high-quality care^(10)^.

Despite the growing adoption of PICC in neonatal care, there remains a significant gap in the literature regarding the clinical validation of ICNP® Terminological Subsets specific to this population. Neonatal nursing practice demands standardized tools that facilitate effective communication among professionals and improve diagnostic accuracy and targeted intervention implementation. However, the lack of studies evaluating the applicability and reliability of ICNP® statements in neonates with PICC hinders their integration into clinical practice and systematic care documentation.

In light of this gap, the present study aims to address this need by clinically validating an ICNP® Terminological Subset for neonates hospitalized in NICU. This initiative seeks to provide a scientific foundation that supports nursing decision-making and contributes to the safety and quality of care, thereby justifying the relevance of this research.

Given the essential role of validation studies, an indispensable step in assessing the applicability of technologies in clinical practice, it is imperative to ensure the accuracy and relevance of the tools used in care decision-making. Accordingly, this study aimed to clinically validate an ICNP® Terminological Subset for neonates with PICC in NICU.

## METHOD

### Study Design and Local

This is a clinical validation study in which case studies were applied following the phases established by the NP. The investigation was conducted in a NICU located in a municipality in the Northeast region of Brazil. Additionally, the study adhered to the guidelines of the Standards for Quality Improvement Reporting Excellence (SQUIRE), as outlined by the Enhancing the Quality and Transparency of Health Research (EQUATOR) initiative, ensuring clarity and rigor in the presentation of results^(11)^.

As the empirical foundation, a previously content-validated terminological subset was employed, comprising 31 ND/NO and 154 NI, organized according to the principles of Betty Neuman’s theoretical model. The construction of these statements was based on data collected through physical examination and analysis of medical records of neonates using PICC. The process also included term verification, manual cross-mapping, statement formulation, and content validation, which was conducted by a panel of expert nurses^(7,9)^.

### Population, Selection Criteria, and Sample

The population included in the clinical validation consisted of 68 neonates with PICC. Sample size was calculated using the formula for finite populations: [ n = \frac{N \cdot Z2 \cdot p \cdot (1 - p)}{(e2 \cdot (N - 1)) + Z^2 \cdot p \cdot (1 - p)} ], where n represents the sample size, N is the total population of neonates hospitalized with PICC over the past year, Z corresponds to the confidence level (1.96 for 95% confidence), p refers to the estimated population proportion (set at 0.5 to maximize variability), and e is the adopted margin of error (10%).

Sample composition followed predefined inclusion criteria: neonates with PICC, hospitalized in the NICU during the data collection period. Exclusion criteria included neonates not using the device, those transferred to other institutions before data collection was completed, and neonates born to adolescent mothers.

The exclusion of neonates born to adolescent mothers was based on ethical guidelines for research involving human subjects, as established by Resolution No. 466/2012 of the Brazilian National Health Council (CNS). This regulation states that minors, including adolescents, do not possess full legal capacity and therefore require a legal guardian to sign the Free and Informed Consent Form (FICF).

After applying the inclusion and exclusion criteria, the final sample consisted of 40 neonates. In addition to the neonates, the sample included four expert nurses (EE1, EE2, EE3, EE4) working in the NICU, who met the following inclusion criteria: specialization in neonatology, active practice in NICU settings, formal training in PICC insertion, and at least two years of practical experience performing the procedure. Nurses on leave or vacation during the study period were excluded.

The literature indicates that there is no strict definition regarding the number of expert nurses required for data collection, allowing flexibility based on the study’s needs and available resources. Therefore, the number of participants was determined by convenience^(12)^.

The selected expert nurses underwent a structured six-hour training program, delivered remotely by the lead researcher over the course of one week. The training covered essential topics for the clinical analysis and validation of the ICNP® terminological subset, including the construction of terminological subsets, application of the Nursing Process, clinical judgment, and diagnostic reasoning based on Betty Neuman’s theory. Participants also received detailed instructions on evaluation criteria and standardization of ND/NO and NI, ensuring consistency in analysis. To minimize bias, the training included practical exercises and clinical case simulations, allowing participants to apply the discussed concepts in practice.

### Data Collection

To achieve the study’s objective, the research was structured and conducted in three distinct phases: 1ª) Application of the Nursing Process in clinical practice, encompassing five essential stages - assessment, diagnosis, planning, implementation, and evaluation of nursing care; 2ª) Structuring of case studies, involving clinical decision-making regarding ND/NO and NI statements; 3ª) Assessment of agreement among expert nurses, to ensure clinical validity and accuracy of the ND/NO and NI statements derived from the case studies.

The study was conducted between October 2024 and February 2025. Data collection from neonates occurred from October to December 2024, while the evaluation phase by expert judges took place between January and February 2025.

Data collection was guided by a protocol structured according to the stages of the Nursing Process, which included monitoring participants from PICC insertion to removal, with weekly evaluations. The research team, led by the principal investigator, conducted and documented the assessments of neonates and developed the case studies based on nursing records.

### First Stage

In the initial phase of the study, data collection and physical examination were conducted by the researcher using a structured instrument based on Betty Neuman’s Systems Model, ensuring a theoretically grounded and systematic approach. This instrument was organized into sections covering sociodemographic data, clinical history, nursing diagnoses, planning, implementation, and evaluation. Additionally, laboratory and imaging tests were analyzed to complement the clinical assessment.

A pilot test was initially conducted, previously approved by the Research Ethics Committee, to verify the adequacy of the data collection instrument. Participants in this pilot test were not included in the final study sample, thereby preserving the integrity of the collected data.

The ND/NO statements were established based on clinical indicators described in the operational definitions of the ICNP® terminological subset. For organizational and accessibility purposes, these statements were pre-systematized in a Google Docs form. In cases where novel clinical situations emerged without preexisting statements, the 2019/2020 version of ICNP® guidelines was used to construct new statements, ensuring the incorporation of terms from the appropriate axes. Furthermore, the ND statements were determined based on clinical aspects observed throughout the data collection process, providing a foundation for a detailed analysis of the participants’ conditions.

The planning and implementation stages were carried out in accordance with the NO statements established for each ND, as defined by the terminological subset. The proposed NI were tailored to meet the identified needs, ensuring a personalized approach aligned with the care objectives and adjusted as necessary.

During the evaluation phase, a second round of data collection and physical examination was conducted, constituting the study’s follow-up assessment. This phase focused on evaluating the previously identified ND statements, allowing for verification of the effectiveness of the interventions and the clinical progression of the participants.

### Second Stage

In the second phase of the study, the researcher developed case studies and formulated ND/NO and NI statements. These data were organized in a Microsoft Excel® spreadsheet, structured into separate tabs, each corresponding to an individual patient.

Subsequently, the statements were categorized according to stressor levels: intrapersonal, interpersonal, and extrapersonal. After this categorization, the spreadsheets containing the organized statements were submitted to four expert nurses for clinical validation.

### Third Stage

In the final phase of the study, the four expert nurses conducted a concordance analysis, evaluating the applicability of the ND/NO and NI statements formulated based on the case studies. Each nurse received a spreadsheet corresponding to one patient, containing all statements organized by stressor level: intrapersonal, interpersonal, and extrapersonal.

In addition to the statements, the spreadsheets included supplementary information such as socioeconomic data, clinical history, and relevant observations for diagnostic inference. The expert nurses individually assessed the presence or absence of ND/NO and NI statements in each spreadsheet, ensuring the clinical accuracy and validity of the terms.

During this phase, ND/NO and NI statements identified in newborns using central venous catheters (CVC) were considered clinically validated, as described in the case studies, and were incorporated into the terminological subset. Throughout the clinical validation process, 25 new ND/NO statements and 22 new NI statements were added, addressing essential aspects of neonatal care. Additionally, 3 ND/NO statements and 66 NI statements from the initial subset were excluded for not meeting validation criteria. As a result, the final subset comprised 53 ND/NO statements and 110 NI statements, all validated with 100% agreement among the expert judges.

The International Council of Nurses (ICN) announced a strategic partnership with the Systematized Nomenclature of Medicine – Clinical Terms (SNOMED CT), aiming to integrate ICNP® into the SNOMED CT clinical terminology.

As part of this process, a manual cross-mapping between the two terminologies was conducted to update the definitions within the terminological subset. To ensure conceptual and terminological precision, the authors enlisted the support of a certified English-language translator, responsible for aligning ICNP® concepts with their equivalent terms in SNOMED CT.

Of the 53 clinically validated ND/NO statements at the conclusion of the study, 39 demonstrated equivalence with SNOMED CT, 14 were classified under the intrapersonal stressor level, 7 under the interpersonal level, and 18 under the extrapersonal level.

### Data Analysis and Treatment

The statistical analysis of this study aimed to assess the level of agreement among expert evaluators and to identify potential differences in the assessments of ND/NO and NI. To measure inter-rater reliability, the Kappa coefficient (κ) was employed a widely recognized metric in clinical validation studies that quantifies agreement beyond chance alone^(13)^.

The Kappa coefficient is essential for ensuring the validity and clinical applicability of diagnostic statements, particularly when multiple evaluators assess identical diagnostic criteria. Agreement levels were classified as follows: poor (0.00–0.19), fair (0.20–0.39), moderate (0.40–0.59), substantial (0.60–0.79), almost perfect (0.80–0.99), and perfect (1.00)^(14)^.

Additionally, to examine statistical differences in the evaluations conducted by the experts, the Kruskal-Wallis test was applied. This non-parametric statistical method is suitable for comparing distributions across three or more independent groups and was selected for its robustness in scenarios where data may not meet normality assumptions, thereby enhancing the precision and reliability of the results. The use of these statistical tools enabled a rigorous evaluation of the clinical validity of the terminological subset, ensuring its consistency and relevance in neonatal care. A significance level of p ≤ 0.05 was adopted.

Furthermore, the Intraclass Correlation Coefficient (ICC) was used to determine the reliability of the terminological subset across different contexts. Reliability was classified as excellent (ICC ≥ 0.75), satisfactory (ICC between 0.40 and 0.75), and poor (ICC < 0.40)^(13)^.

Data related to ND/NO statements were analyzed using simple descriptive statistics, expressed in absolute and relative frequencies. Due to the large number of statements, NI were grouped according to the theoretical model category and discussed based on frequency.

To ensure accuracy in data organization, all information was recorded in an electronic spreadsheet and reviewed twice prior to export into the Statistical Package for the Social Sciences (SPSS), version 20.0. After coding and tabulation, the data underwent statistical and reflective analysis, with the Kappa test applied using the Online Kappa Calculator^(14)^.

In the context of this research, ND/NO and NI statements were considered clinically validated if they met the following criteria: identification in patients and documentation in case studies; inclusion in the Terminological Subset^(15)^; Kappa coefficient classified as almost perfect or perfect; no significant variation in mean evaluations according to the Kruskal-Wallis test; and ICC classified as satisfactory or excellent^(16)^.

### Ethical Aspects

This study was conducted in accordance with ethical principles of voluntariness, anonymity, and confidentiality, following the guidelines of Resolution No. 466/12 of the Brazilian National Health Council (CNS), which regulates research involving human subjects^(17)^. The study was duly approved by the Research Ethics Committee of a federal higher education institution, under approval number 7.171.001, issued in 2024.

To ensure informed and voluntary participation, all individuals involved, including the Free and Informed Consent Form (FICF), thereby ensuring compliance with applicable ethical and scientific standards.

## RESULTS

The study sample consisted of 40 neonates with PICC, distributed across both sexes, with 65% female and 35% male. Gestational age ranged from 26 to 37 weeks, and the time of hospitalization prior to PICC insertion varied between the first and fifth day of admission. Regarding birth weight, 65% of neonates presented with low birth weight, 15% were classified as very low birth weight, 10% as extremely low birth weight, and 10% had weight considered appropriate for gestational age.

All neonates included in the study presented with at least one comorbidity, with respiratory distress being the most prevalent (75%), followed by prematurity (70%). The primary indications for PICC insertion were antibiotic therapy (80%), parenteral nutrition (65%), and administration of vasoactive drugs (15%). The average duration of PICC use was 10.23 days, underscoring its relevance in neonatal therapeutic support.

Regarding the site of PICC insertion, 55% of catheters were placed in the upper limbs. The insertion procedure required an average of 3.72 attempts to achieve success. Post-insertion radiographic verification revealed that 60% of catheters were positioned intracardially, necessitating traction for correction.

Of the 31 ND/NO from the original terminological subset, 28 statements (87%) were implemented, with an average of 16 statements per case study, ranging from a minimum of 9 to a maximum of 28. Three diagnoses, bradycardia, impaired peripheral tissue perfusion, and risk of embolism, were not identified in any case study, preventing their clinical validation in the context of neonatal care with PICC.

In cases where novel clinical situations emerged without preexisting statements, the 2019/2020 version of the ICNP® guidelines was used to construct new statements, ensuring the incorporation of terms from the appropriate axes.

Tables 1, 2, and 3 present the distribution of ND/NO statements according to the stressor levels defined by Betty Neuman’s theoretical model: intrapersonal, interpersonal, and extrapersonal. During analysis, researchers identified the need to include 25 additional ND/NO statements not previously contemplated, resulting in a total of 53 validated nursing diagnoses.

**Table 1:** Agreement of specialist nurses in relation to the nursing diagnosis statements of the level of intrapersonal stressor expressed by the Kappa coefficient. Natal, RN, Brazil, 2025.

Nursing Diagnoses/Outcomes Stressor level: intrapersonal	EE 1	EE 2	EE 3	EE4	Kruskal-Wallis
Bleeding Risk/Bleeding Risk Management	1.00	1.00	0.99	1.00	0.271
Impaired Adaptation/Impaired Adaptation, Absent	1.00	1.00	0.97	1.00	0.137
Impaired Active Range of Motion/Range of Motion, Effective	1.00	1.00	1.00	0.98	0.368
Impaired respiratory system function/Resp. System Function, effective	1.00	1.00	1.00	1.00	0.092
Impaired psychomotor activity/Psychomotor activity, Effective	1.00	0.98	0.96	0.96	0.536
Delayed growth/Delayed growth, Absent	1.00	0.99	0.98	1,00	0.802
Impaired newborn development/NB development, Effective	1.00	1.00	0.99	1.00	0.156
Underweight/Weight within normal limits	1.00	1.00	1.00	1.00	0.214
Impaired nutritional intake/Nutritional intake, Improved	0.98	1.00	1.00	1.00	0.406
Impaired nutritional intake risk/Nutritional intake, Improved	1.00	1.00	1.00	1.00	0.529
Immune impairment/Immune system function, Effective	1.00	0.96	1.00	0.98	0.302
Acid-base imbalance/Acid-base imbalance, Absent	0.98	1.00	0.99	1.00	0.412
Electrolyte imbalance/Electrolyte imbalance, Absent	0.99	1.00	1.00	0.98	0.204
Dehydration/Dehydration, Absent	0.98	1.00	1.00	1.00	0.528

**Table 2:** Agreement of specialist nurses in relation to the nursing diagnosis statements of the level of interpersonal stressor expressed by the Kappa coefficient. Natal, RN, Brazil, 2025.

Nursing Diagnoses/Outcomes Stressor level: interpersonal	EE 1	EE 2	EE 3	EE 4	Kruskal-Wallis
1. Agitation/Agitation, Absent	0.98	0.94	1.00	0.98	0.332
2. Adverse Catheter Location/Catheter Location, Effective	1.00	0.99	1.00	1.00	0.159
3. Interrupted breastfeeding/Breastfeeding, Effective	0.96	1.00	1.00	0.96	0.538
4. Impaired Parenting/Parenting, Effective	0.98	1.00	1.00	0.98	0.364
5. Impaired family process/Family process, Effective	0.99	1.00	1.00	0.99	0.549
6. Interrupted Family Process/Family Process, Improved	1.00	1.00	1.00	0.99	0.320
7. Risk of compromised mother/father and child bond/Absent Risk	1.00	0.98	0.98	1.00	0.134
8. Risk of impaired parental role performance/Absent Risk	1.00	0.96	0.96	1.00	0.450
9. Complex drug regimen/Control	1.00	0.98	0.98	1.00	0.136
10. Risk of healthcare-associated complications/Risk Management	1.00	0.99	0.94	1.00	0.302
11. Risk of cross-infection/Control of risk of cross-infection	1.00	0.99	0.97	1.00	0.142

**Table 3:** Agreement of specialist nurses in relation to the nursing diagnosis statements of the level of extrapersonal stressor expressed by the Kappa coefficient. Natal, RN, Brazil, 2025.

Nursing Diagnoses/Outcomes Stressor level: extrapersonal	EE 1	EE 2	EE 3	EE 4	Kruskal-Wallis
Acute Pain/Pain Management	1.00	0.99	1.00	0.98	0.076
Infection Risk/Infection Risk Control	1.00	1.00	0.98	1.00	0.052
Infection/Infection, Absent	1.00	0.96	1.00	1.00	0.073
Hypothermia/Hypothermia Management	0.98	1.00	1.00	0.98	0.081
Risk of compromised thermoregulation/Risk Control	0.98	1.00	0.98	0.98	0.302
Thermoregulation, Impaired/Thermoregulation, Effective	0.98	1.00	1.00	0.97	0.232
Peripheral edema/Peripheral edema, Absent	0.98	1.00	1.00	0.98	0.702
Hematoma/Hematoma, Absent	1.00	0.99	1.00	0.98	0.304
Skin Integrity, Impaired/Skin Integrity, Effective	1.00	1.00	1.00	1.00	0.101
Invasive Device Trauma/Device Trauma Response, Improved	1.00	1.00	1.00	1.00	0.206
Tissue Integrity, Impaired/Tissue Integrity, Effective	0.96	1.00	0.98	1.00	0.449
Risk of Necrosis/Risk of Necrosis, Absent	1.00	0.99	1.00	1.00	0.502
Function of the Regulatory System, Impaired/Function of the Regulatory System, Effective	1.00	0.98	0.98	0.98	0.302
Vital Sign (Vital Signs), Altered/Vital Sign, within normal limits	1.00	0.98	1.00	1.00	0.512
Risk of Impaired Nervous System Function/Risk Management	0.98	1.00	1.00	0.98	0.074
Risk of Impaired Respiratory System Function/Risk Control	1.00	1.00	1.00	1.00	0.205
Tissue Perfusion Risk, Ineffective/Tissue Perfusion Risk Control	1.00	0.99	0.98	1.00	0.426
Risk of Impaired Neurological Condition/Risk Management	0.99	0.98	0.99	1.00	0.601
Risk of Cardiovascular Function, Impaired/Control of Cardiovascular Function Risk	0.99	1.00	0.99	1.00	0.083
Risk of Heart Rhythm, Impaired/Heart Rhythm, Effective	0.96	1.00	1.00	1.00	0.092
Latex Allergy Risk/Latex Allergy, Controlled	0.98	1.00	0.99	1.00	0.193
Allergy/Allergy, Absent	1.00	0.99	0.98	1.00	0.248
Medication Allergy Risk/Medication Allergy Risk Management	0.97	1.00	1.00	1.00	0.749
Healthcare-associated complication/Complication, Absent	1.00	0.98	1.00	0.96	0.082
Catheter Obstruction/Catheter Obstruction, Absent	1.00	1.00	1.00	1.00	0.163
Risk of Adverse Drug Interaction/Risk of Adverse Drug Interaction, Absent	1.00	1.00	1.00	1.00	0.079
Drug Interaction, Adverse/Adverse Drug Interaction, Absent	1.00	1.00	1.00	1.00	0.831
Risk of impaired child development/Risk management	1.00	1.00	1.00	1.00	0.294

The following ND statements were added under the intrapersonal stressor level: impaired adaptation, impaired active range of motion, impaired respiratory system function, impaired psychomotor activity, delayed growth, impaired neonatal development, underweight, impaired nutritional intake, risk of impaired nutritional intake, immunological compromise, acid-base imbalance, and electrolyte imbalance.

The following ND statements were added under the interpersonal stressor level: adverse catheter placement, interrupted breastfeeding, impaired parenting, compromised family process, interrupted family process, risk of impaired parent-child bonding, risk of impaired parental role performance, complex medication regimen, risk of healthcare-associated complications, and risk of cross-infection.

The following ND statements were added under the extrapersonal stressor level: risk of impaired thermoregulation and risk of impaired infant development, reflecting the influence of environmental and structural factors on neonatal stability.

Regarding the original subset, 66 NI were not clinically validated, while 88 interventions, representing 57% of the total, were approved during clinical validation. Additionally, 22 new NI statements were included to address essential aspects of neonatal care, resulting in a total of 110 validated interventions.

The following NI statements were added: assess gestational age, assess skin integrity, assess risk of apnea, perform physical examination, assess response to pain management, assess response to thermoregulation, assess edema, administer antibiotics, counsel on breastfeeding, support breastfeeding, assess breastfeeding, assess environment, support family, support family decision-making process, ensure continuity of care, assess risk of hospital-acquired complications, care for invasive device insertion site, educate family on infection prevention, educate family on cross-infection prevention, educate family on susceptibility to infection, manage central catheter, and maintain intravenous access.

The inclusion of these interventions reflects the need for comprehensive and specialized approaches in neonatal care with PICC, ensuring safety, complication prevention, and appropriate family support.

Table 4 presents the total number of NI statements identified in the case study participants, along with the corresponding percentage from the original subset. The analysis of ND/NO and NI statements revealed a high level of agreement among the expert evaluators, resulting in the clinical validation of 53 ND/NO and 110 NI statements.

**Table 4:** Nursing interventions identified among case study participants according to stressor levels. Natal, RN, Brasil, 2025.

Stressor Level	N	(%)
Intrapersonal	33	82,5%
Interpersonal	37	92,5%
Extrapersonal	40	100%

The Kruskal-Wallis statistical test demonstrated no significant variation in the mean evaluations conducted by the experts, confirming substantial alignment in their assessments. Additionally, the ICC indicated an excellent reliability level (0.98), ensuring the consistency of the terminological subset and its clinical applicability across diverse neonatal care contexts.

## DISCUSSION

PICCs were initially developed to provide central venous access for the prolonged administration of medications and parenteral nutrition^(18)^. Their extensive use in clinical settings as an alternative to central venous catheters (CVCs) is justified by several advantages, including a lower risk of intraprocedural complications, cost-effective maintenance, feasibility in outpatient care, and a decreased incidence of catheter-related bloodstream infections^(19)^.

In this context, identifying and monitoring stressors affecting neonates with PICCs are essential strategies for promoting health and preventing complications, thereby enhancing both the safety and quality of care provided.

Accordingly, the proposed terminological subset serves as a tool for identifying and articulating individualized care needs. Clear communication, shared responsibilities, and ongoing education are critical components of a successful interprofessional team caring for patients with PICC lines. By employing this instrument, nurses demonstrate professional competence through reliable documentation, which supports continuity and improvement in care delivery.

To this end, the theoretical model adopted played a pivotal role in structuring the subset, guiding it from a nursing perspective with emphasis on the application of stressor levels: intrapersonal, interpersonal, and extrapersonal. Nursing theories are foundational to the development and evaluation of technologies and knowledge, as they themselves represent essential care technologies for implementing best practices in nursing and healthcare^(6)^.

The analysis of stressors faced by neonates with PICCs, through the lens of Neuman’s Systems Model, revealed the multifactorial nature of threats to systemic stability in this population. Intrapersonal, interpersonal, and extrapersonal stressors demonstrated significant interconnection, requiring integrated clinical approaches that are sensitive to the neonatal context.

Clinical validation of ND/NO, and NI based on this theory showed high agreement among expert evaluators, reinforcing the relevance and applicability of the ICNP® terminological subset in the care of neonates with PICCs.

Most statements were classified as extrapersonal stressors, highlighting the predominance of environmental and structural factors within the NICU. Intrapersonal stressors followed, reflecting the biological vulnerability of neonates. Interpersonal stressors were less frequent, possibly due to the technical focus of care in this setting.

The majority of ND/NO statements were deemed appropriate by all four experts, indicating strong consensus regarding their relevance to neonates with PICCs. Terms such as risk of infection and impaired skin integrity were unanimously validated, underscoring concerns about common complications and the importance of comprehensive care.

Interventions also showed a high degree of agreement, although some required semantic and conceptual adjustments to ensure alignment with ICNP® and Betty Neuman’s Systems Model. Critical validation by experienced specialists reinforced the clinical relevance, clarity, and applicability of the terms in neonatal care.

A study focused on the development and validation of ND for premature neonates, based on ICNP®, revealed a high level of agreement among experts throughout the validation process. Of the 146 statements formulated, only one failed to reach a satisfactory content validity index (CVI) for relevance and was therefore excluded from the results^(20)^.

Regarding intrapersonal stressors, the diagnostic statements identified represent stressors directly linked to the individual’s characteristics and conditions^(21)^. These stressors stem from both the physiological immaturity of the neonate and the fragility associated with life-sustaining interventions. Such factors demand a sensitive approach and specialized care strategies to minimize negative impacts on infant well-being.

Intrapersonal stressors, which manifest internally within the neonate’s system, revealed a wide variety and strong interdependence, reflecting the complexity of individual responses to clinical and environmental conditions. Studies indicate that complications related to PICC use include risks of hemorrhage, compromised immune function, thrombosis, and metabolic imbalances such as acid-base and electrolyte disturbances, particularly in extremely premature infants (<32 weeks)^(18,22,23,24)^.

These conditions are often associated with physiological fragility, especially in neonates with birth weight below 1500g or low APGAR scores, increasing susceptibility to infections and metabolic challenges. The rate of catheter-related bloodstream infections was recorded between 10% and 12% of analyzed cases, emphasizing the need for meticulous management and continuous monitoring of PICC^(22,23,24)^.

Nurses’ attention to these stressors is essential for delivering targeted and comprehensive care. By recognizing that these conditions are not merely consequences of the environment or device, but are deeply rooted in the neonate’s physiological and developmental aspects, nurses can tailor interventions to provide more humanized and effective care aligned with the infant’s individual needs. These actions include continuous monitoring, non-pharmacological strategies for pain and discomfort management, support for neuropsychomotor development, and adjustment of nutritional and fluid therapies. Furthermore, early identification and appropriate management of these stressors ensure not only safety but also quality of care, promoting the well-being and healthy development of the neonate.

At the interpersonal stressor level, the DE/RE statements reflected specific responses arising from the direct relationship between the individual and the healthcare professional providing care^(25)^. In the present study, these stressors emerged from the complex interactions between neonates and nurses. Such factors can be significant triggers of stress, particularly in healthcare settings where the relationship between the patient and healthcare providers may influence systemic stability.

During PICC insertion, the interaction between the nurse and the neonate can be a challenging moment, often resulting in discomfort and agitation for the infant. This is due to the need to restrict the baby’s movements to prevent catheter displacement, as well as the inherent pain of the procedure^(26)^. Additionally, incorrect positioning or migration of the PICC may cause further pain and hinder treatment, requiring constant monitoring and adjustments in care. The nursing team must be prepared to manage these situations with sensitivity to ensure the neonate’s comfort and foster parental trust.

Regarding the mother-infant dyad, disruptions to family processes, often necessary due to PICC use, may adversely affect maternal-infant bonding, undermine parental confidence in caring for the baby, and impair the neonate’s nutritional development^(27)^.

Research indicates that breastfeeding support interventions are essential to mitigate the impact of such disruptions. The nursing team plays a crucial role in providing emotional support and information, helping to reduce parental stress. Moreover, efficient and transparent medication management, along with clear communication with parents, can alleviate this burden^(28)^.

At the extrapersonal stressor level, DE/RE statements refer to external influences that may affect the patient’s stability and well-being. These stressors are associated with environmental and circumstantial elements such as policies, healthcare systems, socioeconomic conditions, and available resources. They act indirectly on the neonate’s system and may influence the ability to adapt to other stressors.

Extrapersonal diagnostic statements, such as complications related to healthcare delivery, complex medication regimens, and adverse drug interactions, require constant vigilance to ensure proper treatment administration. The complexity of treatment, combined with the extensive use of medications during PICC therapy, also represents a stressor for the neonate, affecting physiological responses and increasing the likelihood of complications^(29)^.

Based on clinical evaluations, the systematic application of care, exemplified by the use of the terminological subset, supports nurses’ clinical judgment and enables planning tailored to each patient’s specific needs.

Within this framework, nurses conduct clinical reasoning based on patient responses, using ND statements to guide and enhance the care process. The use of standardized languages such as ICNP® allows for the articulation of this knowledge and the implementation of specific NI aligned with patient needs^(13,30,31)^. These interventions focus on continuous team training, education, and behavioral changes, contributing to successful intravenous therapy throughout the neonate’s PICC use^(32,33,34)^.

The impact of the discussed subset on clinical practice is linked to its relevance as a reference for nurses caring for neonates with PICCs in NICUs. This instrument stands out as an effective guide for constructing individualized care plans. Healthcare institutions may adopt such subsets as a strategy to improve care quality, using the statements as references and integrating evidence to support clinical decision-making. These statements can serve as indicators and be transformed into clinical quality metrics^(35)^.

It is noteworthy that the evaluation conducted by professionals using the constructed subset proved highly relevant. In this context, greater consistency in assessments was observed, suggesting improved efficiency in patient analysis. This was made possible by the scientific foundation provided, which validated the indicators used to measure concrete outcomes and supported the development of nursing care plans.

Regarding the study’s limitations, the selection of a specific population may have reduced the generalizability of the findings. Additionally, the study’s restriction to a single region and context introduced a unique cultural characteristic that should be carefully considered when interpreting the results, ensuring a more accurate understanding of the study’s findings.

The implementation of the validated terminological subset for neonates with PICCs in NICUs requires a structured approach integrated into nursing routines. First, ongoing training is essential to equip nurses with the skills to correctly use ICNP® DE/RE and IE statements. Workshops and clinical simulations can help consolidate the clinical reasoning needed to identify indicators of adverse PICC positioning and make informed decisions.

Beyond training, integrating the subset into electronic health record systems can facilitate access to standardized terminology, ensuring greater accuracy in care documentation. The use of clinical decision-support software, offering automated suggestions for diagnoses and interventions based on clinical findings, can optimize practice and reduce inconsistencies in nursing records. This technological integration also enhances interprofessional communication by allowing different members of the healthcare team to access standardized and up-to-date information about patient care.

Another essential factor for the successful implementation of the validated terminological subset is the development of clinical care protocols grounded in the approved definitions. Incorporating these terms into NICU procedural manuals can promote greater consistency in nursing practices and reduce variability in the management of clinical cases. Furthermore, the validation criteria may serve as a foundation for internal audits, enabling the monitoring of adherence to best practices and the identification of opportunities for improvement in care delivery.

Finally, conducting multicenter studies may be a strategic step toward expanding the applicability of the validated subset across different healthcare institutions. Replicating the care model in other NICUs will allow for the evaluation of its effectiveness in diverse contexts, reinforcing its utility in clinical practice. To ensure ongoing updates, it is recommended that professionals involved in the research participate in scientific collaboration networks, where they can share findings and contribute to the evolution of the ICNP® terminological set.

The effective implementation of this subset represents a significant advancement in standardizing specialized neonatal nursing language, strengthening clinical decision-making, enhancing patient safety, and ensuring higher-quality care for neonates with PICCs.

## CONCLUSION

This study demonstrated the clinical validation of a terminological subset comprising 53 ICNP® ND/NO statements and 110 NI statements, specifically designed for the care of neonates with PICCs in NICU. This subset resulted from the clinical validation of 90.3% of the original 31 ND/NO statements and 57% of the 154 NI statements, along with the inclusion of 25 new ND/NO statements and 22 new NI, and the exclusion of 3 ND/NO statements and 66 NI.

The statements showed agreement levels classified as almost perfect and perfect, with excellent overall concordance and no statistically significant differences among expert evaluations. Thus, the development of these empirical statements, combined with the clinical validation process, contributes to tailoring nursing statements to specific populations, providing an effective means for enhanced evaluation of nursing interventions.

Based on the findings of this study, future research may explore the impact of the terminological subset on professional training, investigating how its use can improve nurses’ clinical judgment and contribute to the standardization of neonatal care. Another promising direction is the analysis of improvements in interprofessional communication, examining how the adoption of standardized language can optimize information exchange among nurses, physicians, and other professionals involved in neonatal care—enhancing team integration and the quality of care provided. Such future investigations may further strengthen the applicability of the terminological subset, consolidating its relevance in neonatal nursing and expanding its impact on clinical practice.

## DATA AVAILABILITY

The data used during this study are available from the corresponding author upon reasonable request.
