# Supplementary material for: Clinical validation of the terminology subset for newborns with Peripherally Inserted Central Catheter in Intensive Care
Source: Rev Esc Enferm USP. 2025 Oct 3;59:e20250203. doi: 10.1590/1980-220X-REEUSP-2025-0203en (PMC12503121; doi:10.1590/1980-220X-REEUSP-2025-0203en)
Supplement: Translation

# Translation: Validação clínica do subconjunto terminológico para recém-nascidos com Cateter Central de Inserção Periférica em Terapia Intensiva

**DOI:** 10.1590/1980-220X-REEUSP-2025-0203pt

**Authors:** Nanete Caroline da Costa Prado, Harlon França de Menezes, Romanniny Hévillyn Silva Costa Almino, Isabele Silva dos Santos, Rebecca Stefany da Costa Santos, Sérgio Danillo Santana de Lima Juraci, Dhyanine Morais de Lima Raimundo, Richardson Augusto Rosendo da Silva

**Affiliations:** 1 Universidade Federal do Rio Grande do Norte, Programa de Pós-graduação em Enfermagem, Natal, RN, Brasil.

**Keywords:** Pesquisa em Enfermagem Clínica, Estudo de Validação, Cateterismo Venoso Central, Terminologia Padronizada em Enfermagem, Recém-Nascido

## Abstract

**Objetivo:**

Validar clinicamente um subconjunto terminológico da Classificação Internacional para a Prática de Enfermagem (CIPE®) para recém-nascidos (RN) com Cateter Central de Inserção Periférica (CCIP), em Unidade de Terapia Intensiva Neonatal (UTIN).

**Método:**

Estudo de validação clínica de subconjunto terminológico da CIPE®. A pesquisa foi conduzida por meio das seguintes etapas: aplicação do Processo de Enfermagem em RN internados em uma UTIN, desenvolvimento de estudos de caso e avaliação da concordância entre especialistas. Para a análise dos dados, foram utilizados o coeficiente de concordância de Kappa, o teste de Kruskal-Wallis e o coeficiente de correlação intraclasse (CCI).

**Resultados:**

A avaliação dos diagnósticos/resultados e das intervenções indicou uma concordância variando entre quase perfeita e perfeita, com CCI classificado como excelente. A aplicação do teste de Kruskal-Wallis revelou a ausência de diferenças estatisticamente significativas entre as avaliações.

**Conclusão:**

O estudo validou clinicamente um subconjunto de 53 diagnósticos/resultados e 110 intervenções de enfermagem, demonstrando sua aplicabilidade na assistência a neonatos com Cateter Central de Inserção Periférica.

## INTRODUÇÃO

Atualmente, a incorporação de tecnologias no âmbito do cuidado em saúde revela-se de extrema relevância, por contribuir para uma assistência de maior qualidade e assegurar maior segurança ao paciente, sobretudo em procedimentos invasivos e de elevada complexidade^(1)^. Dentre essas tecnologias, destaca-se o Cateter Central de Inserção Periférica (CCIP), dispositivo indicado para pacientes que necessitam de tratamento prolongado ou que apresentam dificuldades no acesso venoso periférico^(2)^.

A eficácia do CCIP é evidenciada na administração de medicamentos e na nutrição parenteral, configurando-se como uma alternativa segura e eficiente. Dessa forma, garantir um acesso venoso adequado constitui um fator essencial para o êxito do processo de recuperação clínica do paciente^(1,2)^.

O CCIP é amplamente indicado para recém-nascidos, em virtude da facilidade de implantação e dos desafios relacionados à obtenção de acesso venoso periférico nessa faixa etária. Seu uso tem se tornado cada vez mais frequente na assistência neonatal, especialmente em unidades de terapia intensiva neonatal (UTIN). Entre suas principais vantagens, destacam-se: a possibilidade de inserção diretamente no ambiente de cuidado, sem necessidade de deslocamento do paciente; o menor desconforto para o neonato; o risco reduzido de infecção em comparação a outros dispositivos de acesso venoso; o período prolongado de utilização; e a diminuição do estresse provocado por múltiplas punções^(2)^.

A inserção do CCIP deve ser realizada por enfermeiros devidamente capacitados, conforme estabelece a Resolução nº 258/2001 do Conselho Federal de Enfermagem (COFEN). A responsabilidade pela manutenção do dispositivo recai sobre a equipe de enfermagem, com o objetivo de reduzir potenciais complicações e assegurar a segurança do paciente^(3)^.

Diversos cuidados são imprescindíveis, desde o momento da inserção do CCIP, que requer o uso adequado de Equipamentos de Proteção Individual (EPIs) e a aplicação rigorosa da técnica asséptica, até as etapas de manutenção, que incluem a troca de curativos estéreis sempre que necessário, a realização de flushing para prevenção da obstrução do cateter e a desinfecção criteriosa durante a manipulação, com vistas à minimização do risco de infecções^(1,3)^.

Nesse contexto, diversas complicações podem resultar na retirada não eletiva do cateter, frequentemente associadas ao uso de dispositivos venosos. Em um ensaio clínico randomizado realizado com 46 neonatos, observou-se uma taxa de complicações variando entre 30,7% e 62,2%, sendo as mais recorrentes: infecção (14,2%), obstrução do cateter (12%), infiltração (4%) e tração (4%). Em estudo semelhante, conduzido com uma amostra de 111 recém-nascidos, verificou-se uma incidência de complicações em 51% dos casos, destacando-se o posicionamento inadequado (25,7%), flebite (19,3%) e oclusão (3,7%) como as mais prevalentes^(4)^.

O enfermeiro exerce um papel fundamental na elaboração e na execução do cuidado, sendo o Processo de Enfermagem (PE) uma ferramenta estratégica para promover um atendimento mais organizado, sistematizado e eficiente^(5,6)^. O PE é composto por cinco etapas interdependentes e inter-relacionadas: avaliação, diagnóstico, planejamento, implementação e evolução da assistência de enfermagem^(7)^.

Conforme estabelece a Resolução nº 736/2024 do COFEN, é imprescindível que o Processo de Enfermagem esteja alinhado a sistemas de padronização da linguagem, como a Classificação Internacional para a Prática de Enfermagem (CIPE®), a fim de assegurar maior consistência, precisão e qualidade no cuidado prestado^(7,8)^.

A CIPE^®^ desempenha um papel essencial na documentação das ações do enfermeiro na prática clínica, promovendo padronização, organização e maior precisão nos registros assistenciais. Essa padronização favorece uma comunicação clara e eficaz entre os profissionais de saúde, além de facilitar a sistematização da assistência prestada. A CIPE® também viabiliza o desenvolvimento de Subconjuntos Terminológicos, os quais reúnem enunciados de diagnósticos/resultados (DE/RE) e intervenções de enfermagem (IE), direcionados a grupos específicos de pacientes, contribuindo para a personalização e qualificação do cuidado^(9)^.

Dessa forma, a CIPE® pode ser definida como um sistema de comunicação especializado e representativo da prática de enfermagem, com aplicabilidade tanto em contextos locais quanto globais, contribuindo diretamente para a efetividade do Processo de Enfermagem. Trata-se de uma linguagem estruturada que constitui uma estratégia eficiente para a coleta, o armazenamento e a análise de dados em enfermagem, favorecendo o fortalecimento e a valorização da profissão.

Ressalta-se que, para aprimorar a estruturação dos Subconjuntos Terminológicos e ampliar sua eficácia, é imprescindível que esses sejam validados no contexto clínico, assegurando sua aplicabilidade prática e relevância assistencial.

É importante destacar que, para uma estruturação mais robusta dos Subconjuntos Terminológicos e para ampliar sua eficácia, é fundamental a adoção de um modelo teórico ou conceitual que sirva como base para a prática clínica. Dentre os principais referenciais teóricos, destaca-se o modelo de Betty Neuman, que concebe o indivíduo como um sistema dinâmico composto por elementos interligados, reconhecendo a influência simultânea de fatores internos e externos sobre o estado de saúde do paciente^(7,9)^.

Em estudo prévio, foi realizada a construção e a validação de conteúdo de um Subconjunto Terminológico da CIPE®, direcionado a recém-nascidos com CCIP, fundamentado no modelo teórico de Betty Neuman. A elaboração desse subconjunto decorreu da necessidade de padronizar os registros da prática de enfermagem e de aprimorar a qualidade da documentação assistencial em uma UTIN^(7)^.

Posteriormente, identificou-se a necessidade de realizar uma validação clínica, com o objetivo de assegurar a aplicabilidade e a confiabilidade do subconjunto terminológico na prática assistencial. A validação clínica permite verificar se os enunciados desenvolvidos refletem adequadamente as condições reais dos recém-nascidos com CCIP, garantindo que os DE/RE e as IE sejam eficazes no contexto clínico. Esse processo viabiliza ajustes pertinentes, contribuindo para o aprimoramento da precisão das terminologias utilizadas e promovendo um cuidado mais direcionado, seguro e qualificado^(10)^.

Apesar da crescente adoção do CCIP na assistência neonatal, observa-se uma carência significativa na literatura quanto à validação clínica de Subconjuntos Terminológicos específicos para essa população. A prática da enfermagem neonatal exige instrumentos padronizados que favoreçam a comunicação eficaz entre os profissionais, além de aprimorar a precisão diagnóstica e a implementação de intervenções direcionadas. No entanto, verifica-se a ausência de estudos que avaliem a aplicabilidade e a confiabilidade dos enunciados da CIPE® em recém-nascidos em uso de CCIP, o que dificulta sua integração à prática clínica e à documentação sistematizada do cuidado.

Diante da lacuna identificada na literatura, esta pesquisa propõe-se a preencher tal necessidade por meio da validação clínica de um Subconjunto Terminológico da CIPE®, direcionado a neonatos internados em UTIN. A proposta visa oferecer um embasamento científico que fortaleça a tomada de decisão dos profissionais de enfermagem e contribua para a segurança e a qualidade da assistência prestada, justificando, assim, a realização do presente estudo.

Considerando o papel essencial dos estudos de validação, etapa fundamental para avaliar a aplicabilidade de tecnologias no contexto da prática clínica, torna-se imprescindível assegurar a precisão e a relevância dos instrumentos utilizados para a tomada de decisão assistencial. Nesse sentido, o presente estudo teve como objetivo validar clinicamente um subconjunto terminológico da CIPE^®^ para RN com CCIP, em UTIN.

## MÉTODO

### Desenho e Local do Estudo

Trata-se de um estudo de validação clínica, no qual foram aplicados estudos de casos conduzidos de acordo com as fases estabelecidas pelo PE. A investigação foi realizada em uma UTIN situada em um município da região Nordeste do Brasil. Além disso, o estudo seguiu as diretrizes do Standards for *Quality Improvement Reporting Excellence* (SQUIRE), estabelecidas pela iniciativa *Enhancing the Quality and Transparency of Health Research* (EQUATOR), assegurando a qualidade e a clareza na apresentação dos resultados^(11)^.

Como base empírica, utilizou-se um subconjunto terminológico validado em relação ao conteúdo, composto por 31 DE/RE e 154 IE, organizados conforme os princípios do modelo teórico de Betty Neuman. A construção desses enunciados foi fundamentada na coleta de dados por meio de exame físico e na análise dos registros de prontuários de neonatos em uso de CCIP. Além disso, o processo envolveu a verificação dos termos, mapeamento cruzado, elaboração dos enunciados e a validação de conteúdo, realizada por um grupo de juízes composto por enfermeiros especialista^(7,9)^.

### População, Critérios de Seleção e Definição da Amostra

A população incluída na validação clínica consistiu em 68 RN em uso CCIP. Para o cálculo da amostra, foi utilizada a fórmula para populações finitas: [ n = \frac{N \cdot Z2 \cdot (N - 1)) + Z^2 \cdot p \cdot (1 - p)} ] onde “n” representa o tamanho da amostra, “N” é a população total de RN internados com CCIP durante o último ano, “Z” corresponde ao nível de confiança estabelecido (1,96 para 95% de confiança), “p” refere-se à proporção populacional estimada (considerada 0,5 para maior variabilidade), e “e” é a margem de erro adotada (10%).

A composição da amostra seguiu critérios de inclusão previamente definidos: RN em uso de CCIP, hospitalizados em UTIN durante o período destinado à coleta de dados. Quanto aos critérios de exclusão, foram desconsiderados os neonatos que não utilizavam o referido dispositivo, aqueles transferidos para outras instituições antes da conclusão da coleta, bem como RN filhos de mães adolescentes.

A exclusão dos neonatos filhos de mães adolescentes fundamentou-se nas diretrizes éticas para pesquisas envolvendo seres humanos, conforme estabelecido pela Resolução nº 466/2012 do Conselho Nacional de Saúde (CNS). Tal normativa considera que menores de idade, incluindo adolescentes, não possuem plena capacidade civil, sendo necessário um responsável legal para a assinatura do Termo de Consentimento Livre e Esclarecido (TCLE).

Após a aplicação dos critérios mencionados, a amostra final foi composta por 40 RN. Além dos RN, a amostra também incluiu quatro enfermeiros especialistas (EE1, EE2, EE3, EE4) em exercício na UTIN, que atenderam aos seguintes critérios de inclusão: especialização em neonatologia, atuação em UTIN, formação específica para inserção de CCIP e experiência prática igual ou superior a dois anos na realização desse procedimento. Enfermeiros afastados por licença ou férias foram excluídos da pesquisa.

A literatura indica que não há uma definição rígida quanto ao número de enfermeiros especialistas envolvidos na coleta de dados, permitindo flexibilidade conforme a necessidade do estudo e os recursos disponíveis, e, por essa razão, esse número foi definido por conveniência^(12)^.

Os enfermeiros especialistas selecionados para este estudo passaram por um treinamento estruturado com duração total de seis horas, conduzido pela pesquisadora principal, distribuídas ao longo de uma semana. Este ocorreu de forma remota e contemplou conteúdos essenciais para a análise e validação clínica dos enunciados do subconjunto terminológico da CIPE®. Foram abordados temas como a construção de subconjuntos terminológicos, aplicação do PE, julgamento clínico e raciocínio diagnóstico baseado na Teoria de Betty Neuman. Além disso, os participantes receberam instruções detalhadas sobre critérios de avaliação e padronização dos DE/RE e IE, garantindo uniformidade na análise. Para minimizar vieses, o treinamento incluiu exercícios práticos e simulações de casos clínicos, permitindo que os especialistas aplicassem os conceitos discutidos na prática.

### Coleta De Dados

Para alcançar o objetivo estabelecido, o estudo foi estruturado e conduzido em três fases distintas: 1ª) Aplicação do PE à prática clínica, abrangendo cinco etapas essenciais - avaliação, diagnóstico, planejamento, implementação e evolução de Enfermagem; 2ª) Estruturação dos estudos de casos, com a decisão clínica dos enunciados de DE/RE e IE; 3ª) Avaliação da concordância entre os enfermeiros especialistas para garantir a validade clínica e a precisão dos DE/RE e IE, elencados a partir Estudos de Casos.

O estudo foi conduzido entre outubro de 2024 e fevereiro de 2025. A coleta de dados dos RN ocorreu no período de outubro a dezembro de 2024, enquanto a etapa de avaliação pelos juízes foi realizada entre janeiro e fevereiro de 2025.

A coleta de dados foi guiada por um protocolo, estruturado de acordo com as etapas do PE, que estipulou o acompanhamento dos participantes desde a inserção do cateter até sua remoção, com avaliações semanais. A equipe responsável, liderada pela pesquisadora principal, realizou e registrou as avaliações aos RN, bem como estruturaram os estudos de caso mediante os registros de enfermagem.

### Primeira Etapa

Na primeira fase do estudo, a coleta de dados e o exame físico foram conduzidos pela pesquisadora, com o suporte de um instrumento, baseado no Modelo de Betty Neuman, garantindo uma abordagem estruturada e fundamentada na teoria. Esse instrumento foi estruturado em seções que contemplavam dados sociodemográficos, histórico clínico, diagnósticos, planejamento, implementação e avaliação de enfermagem. Além disso, foram analisados exames laboratoriais e de imagem para complementar a avaliação clínica.

Inicialmente foi realizado um teste-piloto, previamente aprovado pelo comitê de ética, com o objetivo de verificar sua adequação do instrumento de coleta. Os participantes desse teste não foram incluídos na amostra final do estudo, garantindo a integridade dos dados coletados.

Os enunciados de DE/RE foram estabelecidos com base em indicadores clínicos descritos nas definições operacionais do subconjunto terminológico da CIPE®. Para organização e acessibilidade, esses enunciados foram previamente sistematizados em um formulário no Google Docs. Nos casos em que surgiram situações clínicas inéditas, sem um enunciado preexistente, foram utilizadas as diretrizes da CIPE® versão 2019/2020 para a construção de novos enunciados, garantindo a incorporação de termos dos eixos correspondentes. Além disso, os DE foram determinados a partir dos aspectos clínicos observados ao longo do processo de coleta de dados, proporcionando subsídios para uma análise detalhada da condição dos participantes.

As etapas de planejamento e implementação foram realizadas em conformidade com os RE estabelecidos para cada DE, conforme definido pelo subconjunto terminológico. As IE propostas foram orientadas para atender às necessidades identificadas, assegurando uma abordagem personalizada e alinhada aos objetivos do cuidado prestado e as devidas adequações quando necessário.

Na etapa de avaliação, foi realizada uma nova coleta de dados e um exame físico, compondo a segunda avaliação do estudo. O foco dessa fase esteve voltado para a avaliação dos enunciados de DE previamente identificados, permitindo verificar a efetividade das intervenções e a evolução clínica dos participantes.

### Segunda Etapa

Na segunda fase do estudo, a pesquisadora desenvolveu os estudos de caso e formulou os enunciados de DE/RE e IE. Essas informações foram organizadas em uma planilha do Microsoft Excel®, estruturada em abas separadas, com cada aba correspondente a um paciente.

Em seguida, os enunciados foram categorizados de acordo com os níveis de estressores: intrapessoal, interpessoal e extrapessoal. Após essa etapa, as planilhas contendo os enunciados organizados foram encaminhadas aos quatro enfermeiros especialistas para o processo de validação clínica.

### Terceira Etapa

Na fase final do estudo, os quatro enfermeiros especialistas realizaram uma análise de concordância, avaliando a aplicabilidade dos enunciados de DE/RE e IE formulados com base nos estudos de caso. Cada enfermeiro recebeu uma planilha referente a um paciente, contendo todos os enunciados organizados conforme os níveis de estressores intrapessoal, interpessoal e extrapessoal.

Além dos enunciados, as planilhas incluíam informações complementares, como dados socioeconômicos, histórico clínico e observações relevantes para o processo de inferência diagnóstica. Os enfermeiros especialistas avaliaram individualmente a presença ou ausência dos DE/RE e IE em cada planilha, assegurando a precisão e validade clínica dos termos.

Durante essa etapa, foram considerados clinicamente validados os DE/RE e IE identificados nos RN em uso de CCIP, conforme descritos nos estudos de caso, os quais foram incorporados ao subconjunto terminológico. No decorrer da validação clínica, foram acrescidos 25 novos DE/RE e 22 novas IE que contemplavam aspectos fundamentais do cuidado neonatal, além da exclusão de 3 DE/RE e 66 IE do subconjunto inicial por não atenderem aos critérios de validação. Como resultado, obteve-se um subconjunto final composto por 53 DE/RE e 110 IE, todos validados com 100% de concordância entre os juízes especialistas.

O Conselho Internacional de Enfermeiros (ICN) anunciou uma parceria estratégica com a *Systematized Nomenclature of Medicine - Clinical Terms* (SNOMED CT), com o objetivo de integrar a CIPE® à terminologia clínica SNOMED CT.

Como parte desse processo, foi realizado um mapeamento cruzado manual entre as duas terminologias, com vistas à atualização das definições do subconjunto terminológico. Para assegurar a precisão conceitual e terminológica, os autores contaram com o suporte de um tradutor juramentado na língua inglesa, responsável por alinhar os conceitos da CIPE® aos termos equivalentes na SNOMED CT.

Dos 53 DE/RE validados clinicamente ao final do estudo, 39 mostraram equivalência com a SNOMED-CT, dos quais 14 estavam classificados no nível de estressor intrapessoal, 7 no nível de estressor interpessoal e 18 no nível extrapessoal.

### Análise e Tratamento dos Dados

A análise estatística do estudo foi conduzida com o objetivo de avaliar a concordância entre os especialistas e verificar possíveis diferenças nas avaliações dos DE/RE e IE. Para mensurar a confiabilidade interavaliadores, foi utilizado o Coeficiente de Kappa (κ), uma medida amplamente reconhecida em estudos de validação clínica, que permite quantificar a concordância entre os especialistas além do que ocorreria por mero acaso^(13)^.

O Kappa é essencial para garantir a validade dos enunciados e sua aplicabilidade na prática clínica, especialmente quando múltiplos avaliadores analisam os mesmos critérios de julgamento diagnóstico. Os níveis de concordância foram classificados da seguinte maneira: pobre (0 a 0,19), relativa (0,20 a 0,39), moderada (0,40 a 0,59), substancial (0,60 a 0,79), quase perfeita (0,80 a 0,99) e perfeita (1,00)^(14)^.

Além disso, para verificar diferenças estatísticas nas avaliações realizadas pelos especialistas, foi aplicado o Teste de Kruskal-Wallis, um método estatístico não paramétrico adequado para comparar distribuições entre três ou mais grupos independentes. Esse teste foi escolhido por sua robustez em situações onde a distribuição dos dados pode não atender aos pressupostos de normalidade, garantindo maior precisão e confiabilidade na análise dos resultados. Assim, a utilização dessas ferramentas estatísticas permitiu uma avaliação criteriosa da validade clínica do subconjunto terminológico, assegurando sua consistência e relevância na assistência neonatal. Adotou-se um nível de significância de p ≤ 0,05.

Adicionalmente, utilizou-se o Coeficiente de Correlação Intraclasse (CCI) para determinar a confiabilidade do subconjunto terminológico em diferentes contextos. A confiabilidade foi classificada como excelente (CCI ≥ 0,75), satisfatória (CCI entre 0,40 e 0,75) e pobre (CCI < 0,40)^(13)^.

Os dados referentes aos enunciados de DE/RE foram analisados por meio de estatísticas descritivas simples, expressas em frequências absoluta e relativa. Devido ao grande número de enunciados, as IE foram agrupadas conforme a categoria do modelo teórico e discutidas com base na frequência.

Para garantir a precisão na organização dos dados, todas as informações foram registradas em uma planilha eletrônica, revisadas duas vezes antes da exportação para o software Statistical Package for Social Science (SPSS) versão 20.0. Após a codificação e tabulação, os dados foram submetidos a uma análise estatística e reflexiva, sendo o teste de Kappa aplicado por meio do Online Kappa Calculator^(14)^.

No contexto desta pesquisa, foram considerados clinicamente validados os enunciados de DE/RE e IE que atenderam aos seguintes critérios: identificação nos pacientes e documentação nos estudos de caso; presença no Subconjunto Terminológico^(15)^; coeficiente Kappa classificado como quase perfeito ou perfeito; ausência de variação significativa nas médias das avaliações realizadas pelos especialistas, conforme o teste de Kruskal-Wallis; e CCI classificado como satisfatória ou excelente^(16)^.

### Aspectos Éticos

O presente estudo foi conduzido em conformidade com os princípios éticos de voluntariedade, anonimato e confidencialidade, em conformidade com as diretrizes da Resolução nº 466/12 do CNS, que normatiza pesquisas envolvendo seres humanos^(17)^. O estudo foi devidamente aprovado pelo Comitê de Ética em Pesquisa de uma instituição federal de ensino superior, conforme parecer nº 7.171.001, emitido no ano de 2024.

Para garantir a adesão voluntária e esclarecida ao estudo, todos os participantes, incluindo os responsáveis pelos neonatos e enfermeiros especialistas assinaram o TCLE, assegurando a conformidade com as normativas éticas e científicas aplicáveis.

## RESULTADOS

A amostra do estudo foi composta por 40 RN em uso do CCIP, distribuídos entre ambos os sexos, sendo 65% do sexo feminino e 35% do sexo masculino. A idade gestacional dos neonatos variou entre 26 e 37 semanas, enquanto o tempo de internação antes da inserção do CCIP ocorreu entre o primeiro e o quinto dia de hospitalização. Em relação ao peso ao nascer, 65% dos RN apresentaram baixo peso, 15% foram classificados como muito baixo peso, 10% como extremo baixo peso e 10% tinham peso considerado normal.

Todos os RN incluídos no estudo apresentavam alguma comorbidade, sendo o desconforto respiratório a mais prevalente, ocorrendo em 75% dos casos, seguido da prematuridade, observada em 70% dos participantes. As principais indicações para a inserção do CCIP foram antibioticoterapia, necessária em 80% dos casos, nutrição parenteral, aplicada em 65% dos neonatos, e administração de drogas vasoativas, que representou 15% das indicações. A média de permanência do CCIP foi de 10,23 dias, destacando sua relevância no suporte terapêutico neonatal.

Quanto ao local de inserção do CCIP, 55% dos cateteres foram posicionados nos membros superiores. O procedimento de inserção demandou uma média de 3,72 tentativas para obtenção de sucesso. Após a radiografia para verificar a localização da ponta do cateter, constatou-se que 60% estavam em posição intracardíaca, tornando necessário o tracionamento para correção.

Em relação aos 31 DE/RE do subconjunto original, 28 enunciados (87%) foram implementados, com média de 16 enunciados por estudo de caso, variando entre um mínimo de nove e um máximo de 28. Três diagnósticos do subconjunto original, Bradicardia, Perfusão Tissular Periférica Prejudicada e Risco de Embolia, não foram identificados em nenhum dos estudos de caso, o que impediu sua validação clínica no contexto dos cuidados a neonatos com CCIP.

Ressalta-se que nos casos em que surgiram situações clínicas inéditas, sem um enunciado preexistente, foram utilizadas as diretrizes da CIPE® versão 2019/2020 para a construção de novos enunciados, garantindo a incorporação de termos dos eixos correspondentes.

As Tabelas 1, 2 e 3 apresentam a distribuição dos DE/RE identificados conforme os níveis de estressores do modelo de Betty Neuman: intrapessoal, interpessoal e extrapessoal. Durante a análise, os pesquisadores detectaram a necessidade de incluir 25 DE/RE adicionais, que não estavam previamente contemplados, totalizando ao final da validação clínica 53 DE.

**Tabela 1:** Concordância dos enfermeiros especialistas em relação aos enunciados de diagnósticos de enfermagem do Nível de estressor intrapessoal expressado pelo coeficiente de Kappa. Natal, RN, Brasil, 2025.

Diagnósticos/Resultados de Enfermagem Nível de estressor: intrapessoal	EE 1	EE 2	EE 3	EE 4	Kruskal-Wallis
Risco de Hemorragia/Controle do Risco de Hemorragia	1,00	1,00	0,99	1,00	0,271
Adaptação prejudicada/Adaptação prejudicada, Ausente	1,00	1,00	0,97	1,00	0,137
Amplitude de movimento ativo prejudicada/Amplitude do movimento, Eficaz	1,00	1,00	1,00	0,98	0,368
Função do sistema respiratório prejudicada/Função do sist. Resp. comp., Eficaz	1,00	1,00	1,00	1,00	0,092
Atividade psicomotora prejudicada/Atividade psicomotora, Eficaz	1,00	0,98	0,96	0,96	0,536
Crescimento atrasado/Crescimento atrasado, Ausente	1,00	0,99	0,98	1,00	0,802
Desenvolvimento prejudicado do recém-nascido/ Desenvolvimento do RN, Eficaz	1,00	1,00	0,99	1,00	0,156
Abaixo do Peso/Peso dentro dos limites normais	1,00	1,00	1,00	1,00	0,214
Ingestão nutricional prejudicada/Ingestão nutricional, Melhorada	0,98	1,00	1,00	1,00	0,406
Risco de ingestão nutricional prejudicada/Ingestão nutricional, Melhorada	1,00	1,00	1,00	1,00	0,529
Comprometimento imunológico/Função do sist. imunológico, Eficaz	1,00	0,96	1,00	0,98	0,302
Desequilíbrio ácido-base/Desequilíbrio ácido-base, Ausente	0,98	1,00	0,99	1,00	0,412
Desequilíbrio eletrolítico/Desequilíbrio eletrolítico, Ausente	0,99	1,00	1,00	0,98	0,204
Desidratação/Desidratação, Ausente	0,98	1,00	1,00	1,00	0,528

**Tabela 2:** Concordância dos enfermeiros especialistas em relação aos enunciados de diagnósticos de enfermagem do Nível de estressor interpessoal expressado pelo coeficiente de Kappa. Natal, RN, Brasil, 2025.

Diagnósticos/Resultados de Enfermagem Nível de estressor: interpessoal	EE 1	EE 2	EE 3	EE 4	Kruskal-Wallis
1. Agitação/Agitação, Ausente	0,98	0,94	1,00	0,98	0,332
2. Localização Adversa do Cateter/Localização do Cateter, Eficaz	1,00	0,99	1,00	1,00	0,159
3. Amamentação interrompida/Amamentação, Eficaz	0,96	1,00	1,00	0,96	0,538
4. Parentalidade prejudicada/Parentalidade, Eficaz	0,98	1,00	1,00	0,98	0,364
5. Processo familiar comprometido/Processo familiar, Eficaz	0,99	1,00	1,00	0,99	0,549
6. Processo familiar interrompido/Processo familiar, Melhorado	1,00	1,00	1,00	0,99	0,320
7. Risco de ligação mãe/pai e filho comprometida/Risco Ausente	1,00	0,98	0,98	1,00	0,134
8. Risco de desempenho do papel parental comprometido/Risco Ausente	1,00	0,96	0,96	1,00	0,450
9. Regime medicamentoso complexo/Controle	1,00	0,98	0,98	1,00	0,136
10. Risco de complicação associada aos cuidados de saúde/Controle do Risco	1,00	0,99	0,94	1,00	0,302
11. Risco de infeção cruzada/Controle do Risco de infeção cruzada	1,00	0,99	0,97	1,00	0,142

**Tabela 3:** Concordância dos enfermeiros especialistas em relação aos enunciados de diagnósticos de enfermagem do Nível de estressor extrapessoal expressado pelo coeficiente de Kappa. Natal, RN, Brasil, 2025.

Diagnósticos/Resultados de Enfermagem Nível de estressor: extrapessoal	EE 1	EE 2	EE 3	EE 4	Kruskal-Wallis
Dor aguda/Controle da Dor	1,00	0,99	1,00	0,98	0,076
Risco de Infecção/Controle de Risco de Infecção	1,00	1,00	0,98	1,00	0,052
Infecção/Infecção, Ausente	1,00	0,96	1,00	1,00	0,073
Hipotermia/Controle da Hipotermia	0,98	1,00	1,00	0,98	0,081
Risco de termorregulação comprometida/Controle do Risco	0,98	1,00	0,98	0,98	0,302
Termorregulação, Prejudicada/Termorregulação, Eficaz	0,98	1,00	1,00	0,97	0,232
Edema periférico/Edema periférico, Ausente	0,98	1,00	1,00	0,98	0,702
Hematoma/Hematoma, Ausente	1,00	0,99	1,00	0,98	0,304
Integridade da Pele, Prejudicada/Integridade da Pele, Eficaz	1,00	1,00	1,00	1,00	0,101
Trauma por Dispositivo Invasivo/Resposta ao Trauma por Dispositivo, Melhorada	1,00	1,00	1,00	1,00	0,206
Integridade Tissular, Prejudicada/Integridade Tissular, Eficaz	0,96	1,00	0,98	1,00	0,449
Risco de Necrose/Risco de Necrose, Ausente	1,00	0,99	1,00	1,00	0,502
Função do Sistema Regulatório, Prejudicada/Função do Sist. Regulatório, Eficaz	1,00	0,98	0,98	0,98	0,302
Sinal Vital (Sinais Vitais), Alterado/Sinal Vital, no limite normal	1,00	0,98	1,00	1,00	0,512
Risco de Função do Sistema Nervoso, Prejudicada/Controle do Risco	0,98	1,00	1,00	0,98	0,074
Risco de Função do Sistema Respiratório, Prejudicada/Controle do Risco	1,00	1,00	1,00	1,00	0,205
Risco de Perfusão Tissular, Ineficaz/Controle do Risco de Perfusão Tissular	1,00	0,99	0,98	1,00	0,426
Risco de Condição Neurológica, Prejudicada/Controle do Risco	0,99	0,98	0,99	1,00	0,601
Risco de Função Cardiovascular, Prejudicada/Controle do Risco de Função Cardiovascular	0,99	1,00	0,99	1,00	0,083
Risco de Ritmo Cardíaco, Prejudicado/Ritmo Cardíaco, Eficaz	0,96	1,00	1,00	1,00	0,092
Risco de Alergia a Látex/Alergia a Látex, Controlada	0,98	1,00	0,99	1,00	0,193
Alergia/Alergia, Ausente	1,00	0,99	0,98	1,00	0,248
Risco de Alergia a Medicação/Controle do Risco de Alergia a Medicação	0,97	1,00	1,00	1,00	0,749
Complicação associada aos cuidados de saúde/Complicação, Ausente	1,00	0,98	1,00	0,96	0,082
Obstrução do Cateter/Obstrução do Cateter, Ausente	1,00	1,00	1,00	1,00	0,163
Risco de Interação Medicamentosa Adversa/Risco de Interação Medicamentosa Adversa, Ausente	1,00	1,00	1,00	1,00	0,079
Interação Medicamentosa, Adversa/Interação Medicamentosa Adversa, Ausente	1,00	1,00	1,00	1,00	0,831
Risco de desenvolvimento infantil prejudicado/Controle do risco	1,00	1,00	1,00	1,00	0,294

No nível de estressor intrapessoal, foram adicionados DE como: adaptação prejudicada, amplitude de movimento ativo prejudicada, função do sistema respiratório prejudicada, atividade psicomotora prejudicada, crescimento atrasado, desenvolvimento prejudicado do recém-nascido, abaixo do peso, ingestão nutricional prejudicada, risco de ingestão nutricional prejudicada, comprometimento imunológico, desequilíbrio ácido-base e desequilíbrio eletrolítico.

Já no nível de estressor interpessoal, foram identificados e acrescentados os seguintes DE: localização adversa do cateter, amamentação interrompida, parentalidade prejudicada, processo familiar comprometido, processo familiar interrompido, risco de ligação mãe/pai e filho comprometida, risco de desempenho do papel parental comprometido, regime medicamentoso complexo, risco de complicação associada aos cuidados de saúde e risco de infecção cruzada.

Por fim, no nível extrapessoal, foram adicionados os seguintes DE: risco de termorregulação comprometida e risco de desenvolvimento infantil prejudicado, refletindo a influência dos fatores ambientais e estruturais sobre a estabilidade do recém-nascido.

Em relação ao Subconjunto original, 66 IE não foram validadas clinicamente, enquanto 88, equivalentes a 57% do total, foram aprovadas na validação clínica. Durante a análise, identificou-se a necessidade de incluir 22 novas IE que contemplassem aspectos fundamentais do cuidado neonatal, totalizando 110.

Foram acrescentadas 22 IE, a saber: avaliar idade gestacional, avaliar integridade da pele, avaliar risco de apneia, exame físico, avaliar evolução da resposta à gestão da dor, avaliar evolução da resposta à termorregulação, avaliar edema, administrar antibiótico, aconselhar sobre amamentação, apoiar amamentação, avaliar amamentação, avaliar ambiente, apoiar família, apoiar processo de tomada de decisão familiar, assegurar continuidade de cuidados, avaliar risco de complicação adquirida no hospital, cuidados ao local de inserção de dispositivo invasivo, ensinar família sobre prevenção de infecção, ensinar família sobre prevenção de infecção cruzada, ensinar família sobre suscetibilidade à infecção, gerir cateter central e manter acesso intravenoso.

A inclusão dessas intervenções reflete a necessidade de abordagens abrangentes e especializadas no cuidado aos neonatos com CCIP, garantindo segurança, prevenção de complicações e suporte familiar adequado.

A Tabela 4 apresenta o número total de IE identificadas nos participantes dos estudos de caso, assim como a porcentagem correspondente ao subconjunto original. A análise dos DE/RE e IE evidenciou uma concordância elevada entre os especialistas, classificando 53 DE/RE e 110 IE como válidos clinicamente.

**Tabela 4:** Intervenções de enfermagem identificadas nos participantes acompanhados nos estudos de caso, de acordo com os níveis de estressores. Natal, RN, Brasil, 2025.

Nível de estressor	N	(%)
Intrapessoal	33	82,5%
Interpessoal	37	92,5%
Extrapessoal	40	100%

O teste estatístico de Kruskal-Wallis demonstrou que não houve variação significativa nas médias das avaliações realizadas pelos especialistas, confirmando um alinhamento substancial entre suas percepções. Além disso, o CCI indicou um nível de fidedignidade excelente (0,98), garantindo a confiabilidade do subconjunto e sua aplicabilidade clínica em diferentes contextos de cuidado neonatal.

## DISCUSSÃO

Os CCIP foram introduzidos com o propósito de estabelecer acesso venoso central para a administração prolongada de medicamentos e nutrição parenteral^(18)^. Sua ampla utilização na prática clínica como alternativa aos cateteres venosos centrais (CVC) fundamenta-se em diversos fatores, tais como: menor risco de complicações intraprocedimentais, viabilidade econômica para manutenção, funcionalidade em contextos ambulatoriais e redução da incidência de infecções da corrente sanguínea associadas ao cateter^(19)^.

Nesse contexto, a identificação e o monitoramento dos estressores envolvidos no cuidado ao RN em uso de CCIP constituem estratégias relevantes para a promoção da saúde e a prevenção de complicações, contribuindo para o fortalecimento da segurança e da qualidade da assistência prestada.

Destarte, o subconjunto terminológico proposto configura-se como uma ferramenta para a identificação e expressão de necessidades de cuidados individualizadas. Comunicação clara, responsabilidades compartilhadas e educação contínua são componentes críticos de uma equipe interprofissional bem-sucedida cuidando de pacientes com linhas CCIP. Consequentemente, ao utilizar esse instrumento, o enfermeiro demonstra sua competência profissional por meio de registros confiáveis, os quais promovem a continuidade e o aperfeiçoamento da assistência prestada.

Para esse propósito, o modelo teórico adotado desempenhou um papel fundamental na estruturação do subconjunto, orientando-o sob a perspectiva da enfermagem, com ênfase na aplicação dos níveis de estressores: intrapessoal, interpessoal e extrapessoal. Destaca-se que as teorias de enfermagem servem como alicerces fundamentais para a criação e avaliação de tecnologias e conhecimentos, pois, por si só, representam tecnologias do cuidado indispensáveis à implementação de boas práticas na enfermagem e saúde^(6)^.

A análise dos estressores enfrentados por RN com CCIP, à luz da Teoria de Neuman, revelou a natureza multifatorial das ameaças à estabilidade sistêmica dessa população. Os estressores intrapessoais, interpessoais e extrapessoais demonstraram uma interconexão significativa, exigindo abordagens clínicas integradas e sensíveis ao contexto neonatal.

A validação clínica dos DE/RE e IE fundamentados nessa teoria evidenciou uma alta concordância entre os EE, reforçando a pertinência e aplicabilidade do subconjunto terminológico da CIPE® no cuidado ao RN com CCIP.

A maioria dos enunciados foi classificada como estressores extrapessoais, evidenciando a predominância dos fatores ambientais e estruturais da UTIN. Em seguida, destacaram-se os estressores intrapessoais, relacionados à vulnerabilidade biológica dos neonatos. Já os estressores interpessoais apresentaram menor frequência, possivelmente devido ao foco no cuidado técnico nesse contexto assistencial.

A maioria dos DE/RE foi considerada adequada pelos quatro especialistas, evidenciando um consenso sólido quanto à sua pertinência para neonatos com CCIP. Os termos como risco de infecção e integridade da pele prejudicada foram validados unanimemente, ressaltando a preocupação com complicações comuns e a promoção do cuidado integral.

As intervenções também apresentaram um alto grau de concordância, embora alguns termos tenham exigido ajustes semânticos e conceituais para garantir alinhamento com a CIPE® e com o Modelo de Sistemas de Betty Neuman. A validação crítica conduzida por especialistas experientes reforçou a relevância clínica, a clareza e a aplicabilidade dos termos no cenário neonatal.

Uma pesquisa voltada para a construção e validação de DE destinados a RN prematuros, baseada na CIPE®, revelou um alto nível de concordância entre os especialistas ao longo do processo de validação. Dos 146 enunciados formulados, apenas um não alcançou um índice de validade de conteúdo (IVC) satisfatório em relação à relevância, sendo, por esse motivo, excluído dos resultados^(20)^.

Acerca do nível de estressor intrapessoal, os enunciados diagnósticos levantados representam estressores intrapessoais diretamente ligados às características e condições próprias do indivíduo^(21)^. Esses estressores são consequência tanto da imaturidade fisiológica do organismo do RN quanto da fragilidade diante das intervenções necessárias à sua sobrevivência. Esses fatores exigem uma abordagem sensível e estratégias de cuidado especializadas para minimizar impactos negativos no bem-estar infantil.

Os estressores intrapessoais, que se manifestam internamente no próprio sistema do RN, revelaram uma ampla variedade e uma forte interdependência, refletindo a complexidade das respostas individuais frente às condições clínicas e ambientais. Estudos apontam que complicações relacionadas ao uso do CCIP incluem risco de hemorragias, função imunológica comprometida, tromboses e desequilíbrios metabólicos como desequilíbrio ácido-base e eletrolítico, sobretudo em prematuros extremos (<32 semanas)^(18,22-24)^.

Essas condições estão frequentemente ligadas à fragilidade fisiológica, especialmente daqueles com peso ao nascer inferior a 1500g ou APGAR baixo, o que aumenta a suscetibilidade a infecções e dificuldades metabólicas. A taxa de infecção de corrente sanguínea associada ao cateter foi registrada entre 10% e 12% dos casos analisados, evidenciando a necessidade de uma gestão criteriosa e do monitoramento constante do CCIP. Esses cuidados são essenciais para reduzir riscos e garantir a segurança do paciente durante a terapia intravenosa^(22-24)^.

A atenção do enfermeiro a esses estressores é fundamental para promover um cuidado direcionado e integral. Ao compreender que essas condições não são apenas consequências do ambiente ou do dispositivo, mas também estão profundamente enraizadas nos aspectos fisiológicos e no desenvolvimento do neonato, o enfermeiro pode ajustar suas intervenções para proporcionar um cuidado mais humanizado e eficaz, alinhado às necessidades individuais do RN. Essas ações incluem a monitorização contínua, o uso de estratégias não farmacológicas para controle de dor e desconforto, o apoio ao desenvolvimento neuropsicomotor e o ajuste de terapias nutricionais e fluidoterápicas. Além disso, a identificação precoce e o manejo adequado desses estressores garantem não apenas a segurança, mas também a qualidade do cuidado, promovendo o bem-estar e o desenvolvimento saudável do RN.

No nível de estressor interpessoal os enunciados de DE/RE retrataram respostas específicas da relação direta que envolve o indivíduo e o profissional que assiste o paciente^(25)^. Na presente pesquisa, esses estressores surgiram das interações complexas entre os RN e enfermeiros. Tais fatores podem ser desencadeadores significativos de estresse, especialmente em contextos de cuidados de saúde, onde o relacionamento entre o cliente e os profissionais de saúde, pode influenciar a estabilidade do sistema.

Durante a inserção do CCIP, a interação entre o enfermeiro e o neonato pode ser um momento desafiador para o RN, gerando desconforto e agitação. Isso ocorre devido à necessidade de restringir os movimentos do bebê para evitar o deslocamento do cateter, além da dor inerente ao procedimento^(26)^. Além disso, a localização incorreta ou a migração do CCIP pode provocar dor adicional e dificultar o tratamento, exigindo monitoramento constante e ajustes no cuidado. A equipe de enfermagem deve estar preparada para lidar com essas situações com sensibilidade para garantir o conforto do RN e a confiança dos pais.

No tocante a interação envolvendo o binômio mãe-filho, a interrupção dos processos familiares, frequentemente necessária devido ao uso do CCIP, pode ter efeitos adversos no vínculo entre mãe e filho, afetar a confiança dos pais em sua capacidade de cuidar do bebê, além de prejudicar o desenvolvimento nutricional do neonato^(27)^.

Estudo mostra que intervenções de apoio à amamentação são fundamentais para minimizar o impacto dessa interrupção. A equipe de enfermagem tem um papel crucial em oferecer suporte emocional e informações, ajudando a reduzir o estresse parental. Além disso, o gerenciamento eficiente e transparente dos medicamentos, juntamente com a explicação clara para os pais, pode reduzir essa carga de estresse^(28)^.

No nível de estressor extrapessoal os enunciados de DE/RE referem-se a influências externas ao paciente que podem afetar sua estabilidade e bem-estar^(25)^. Esses estressores estão associados a elementos ambientais e circunstanciais, como políticas, sistemas de saúde, condições socioeconômicas, e recursos disponíveis. Eles agem indiretamente sobre o sistema do neonato e podem influenciar a capacidade de adaptação a outros estressores.

Enunciados diagnósticos extrapessoais como as complicações associadas a atenção à saúde, regime medicamentoso complexo, interação medicamentosa adversa exigem uma vigilância constante para garantir que o tratamento esteja administrado corretamente. O caráter complexo do tratamento, somado à ampla administração de medicamentos durante o uso do CCIP, também representa um fator de estresse para o neonato, afetando sua resposta fisiológica e aumentando a probabilidade de complicações^(29)^.

Com base nas avaliações clínicas realizadas, a aplicação sistematizada do cuidado, exemplificada pelo uso do subconjunto, subsidia o julgamento clínico do enfermeiro e possibilita um planejamento alinhado às particularidades de cada paciente.

Diante da temática proposta, o enfermeiro, em sua atuação profissional, conduz o julgamento clínico com base na análise das respostas do paciente, empregando DE para direcionar e qualificar o processo de cuidado. O emprego de linguagens padronizadas, como a CIPE®, permite descrever esse conhecimento e aplicar IE específicas de acordo com as necessidades do paciente^(13,30,31)^. Tais intervenções focam no treinamento contínuo da equipe, educação, e mudanças comportamentais, contribuindo para o alcance do sucesso durante toda a terapia endovenosa ao neonato envolvendo o uso do CCIP como dispositivo de escolha^(32-34)^.

O impacto do subconjunto discutido na prática assistencial está vinculado à sua relevância como referência para enfermeiros que prestam cuidados a neonatos em uso de CCIP em UTIN. Este instrumento se destaca como um guia eficaz para a construção de planos de cuidado individualizados. As instituições de saúde podem adotar subconjuntos como uma estratégia para elevar a qualidade da assistência, empregando os enunciados como referência e integrando evidências para fundamentar a tomada de decisão clínica, aprimorar a assistência, utilizando os enunciados como indicativos e incorporando evidências retornadas que podem ser convertidas em indicadores clínicos de qualidade^(35)^.

Destaca-se que a avaliação realizada pelos profissionais ao utilizarem o subconjunto construído se mostrou de grande relevância. Nesse contexto, observou-se uma maior homogeneidade nas avaliações, o que sugere uma maior eficiência na análise dos pacientes. Isso foi possível devido à fundamentação científica proporcionada, que validou os indicadores utilizados para mensurar resultados concretos, proporcionando suporte à elaboração do plano de cuidados de enfermagem.

No que diz respeito as limitações do estudo, a seleção de uma população específica pode ter reduzido a abrangência dos achados, tornando sua generalização mais desafiadora. Além disso, o fato de o estudo estar restrito a uma única região e contexto introduziu uma característica cultural singular, que deve ser considerado atentamente ao analisar os resultados, garantindo uma compreensão mais precisa dos achados do estudo.

A implementação do subconjunto terminológico validado para neonatos com CCIP na UTIN exige uma abordagem estruturada e integrada à rotina dos profissionais de enfermagem. Primeiramente, é fundamental promover treinamentos contínuos para capacitar enfermeiros na correta utilização dos enunciados de DE/RE e IE da CIPE®. A realização de workshops e simulações clínicas pode ajudar a consolidar o raciocínio clínico necessário para identificar os indicadores específicos do diagnóstico de localização adversa do CCIP e tomar decisões assertivas.

Além do treinamento, a integração do subconjunto nos sistemas eletrônicos de prontuário pode facilitar o acesso dos profissionais às terminologias padronizadas, garantindo maior precisão na documentação do cuidado. O uso de softwares de suporte à decisão clínica, com sugestões automatizadas de diagnósticos e intervenções baseadas nos achados clínicos, pode otimizar a prática e minimizar inconsistências nos registros de enfermagem. Essa integração tecnológica também favorece a comunicação interprofissional, ao permitir que diferentes membros da equipe de saúde acessem informações padronizadas e atualizadas sobre o cuidado prestado ao paciente.

Outro aspecto essencial para a implementação bem-sucedida do subconjunto terminológico é a criação de protocolos assistenciais baseados nas definições validadas. A incorporação desses termos nos manuais de conduta da UTIN pode garantir maior uniformidade nas práticas de enfermagem e reduzir variações na abordagem dos casos clínicos. Além disso, os critérios de validação podem servir como base para auditorias internas, permitindo monitorar a adesão às melhores práticas e identificar oportunidades de melhoria no cuidado prestado.

Por fim, a realização de estudos multicêntricos pode ser um passo estratégico para ampliar a aplicabilidade do subconjunto validado em diferentes instituições de saúde. A replicação do modelo assistencial em outras UTIN permitirá avaliar sua eficácia em diversos contextos, reforçando sua utilidade na prática clínica. Para garantir a continuidade da atualização, recomenda-se que os profissionais envolvidos na pesquisa participem de redes de colaboração científica, onde possam compartilhar achados e contribuir para a evolução do conjunto terminológico da CIPE®.

A implementação eficaz desse subconjunto representa um avanço significativo na padronização da linguagem especializada da enfermagem neonatal, fortalecendo a tomada de decisão dos profissionais, aprimorando a segurança do paciente e garantindo uma assistência mais qualificada aos recém-nascidos em uso de CCIP.

## CONCLUSÃO

A pesquisa evidenciou a validação clínica de um subconjunto composto por 53 DE/RE e 110 IE da CIPE®, direcionado ao cuidado de RN em uso de CCIP na UTIN. Esse subconjunto resulta da validação clínica de 90,3% dos 31 DE/RE e 57% das 154 IE originalmente presentes no subconjunto terminológico inicial, além da inclusão de 25 novos DE/RE e 22 novas IE e da exclusão de 3 DE/RE e 66 IE.

Os enunciados demonstraram índices de concordância classificados como quase perfeita e perfeita, além de uma concordância global excelente, sem evidências de diferenças estatisticamente significativas entre as avaliações realizadas pelos especialistas. Dessa forma, a elaboração desses enunciados empíricos, aliada ao processo de validação clínica, contribui para a adequação dos enunciados de enfermagem a populações específicas, proporcionando um meio eficaz para a avaliação aprimorada das intervenções de enfermagem.

Diante dos achados deste estudo, futuras pesquisas podem explorar o impacto do subconjunto terminológico na capacitação de profissionais, investigando como seu uso pode aprimorar o julgamento clínico dos enfermeiros e contribuir para a padronização da assistência neonatal. Outra direção promissora é a análise da melhoria na comunicação interprofissional, verificando como a adoção de uma linguagem padronizada pode otimizar a troca de informações entre enfermeiros, médicos e demais profissionais envolvidos no cuidado neonatal, potencializando a integração das equipes e a qualidade da assistência prestada. Essas investigações futuras poderão fortalecer ainda mais a aplicabilidade do subconjunto terminológico, consolidando sua relevância na enfermagem neonatal e ampliando seu impacto na prática clínica.

## DISPONIBILIDADE DE DADOS

Os dados utilizados durante este estudo estão disponíveis com o autor correspondente mediante solicitação razoável.

# Translation: Validación clínica del subconjunto terminológico para recién nacidos con Catéter Central de Inserción Periférica en Cuidados Intensivos

**DOI:** 10.1590/1980-220X-REEUSP-2025-0203es

**Authors:** Nanete Caroline da Costa Prado, Harlon França de Menezes, Romanniny Hévillyn Silva Costa Almino, Isabele Silva dos Santos, Rebecca Stefany da Costa Santos, Sérgio Danillo Santana de Lima Juraci, Dhyanine Morais de Lima Raimundo, Richardson Augusto Rosendo da Silva

**Affiliations:** 1 Universidade Federal do Rio Grande do Norte, Programa de Pós-graduação em Enfermagem, Natal, RN, Brasil.

**Keywords:** Investigación en Enfermería Clínica, Estudio de Validación, Cateterismo Venoso Central, Terminología Normalizada de Enfermería, Recién Nacido

## Abstract

**Objetivo:**

Validar clínicamente un subconjunto terminológico de la Clasificación Internacional para la Práctica de Enfermería (CIPE®) para recién nacidos (RN) con Catéter Central de Inserción Periférica (PICC), en Unidad de Cuidados Intensivos Neonatales (UCIN).

**Método:**

Estudio de validación clínica del subconjunto de terminología de la CIPE®. La investigación se llevó a cabo a través de las siguientes etapas: aplicación del Proceso de Enfermería en recién nacidos ingresados en una UCIN, desarrollo de estudios de casos y evaluación de concordancia entre expertos. Para el análisis de los datos se utilizó el coeficiente de concordancia Kappa, la prueba de Kruskal-Wallis y el coeficiente de correlación intraclase (CCI).

**Resultados:**

La evaluación de los diagnósticos/resultados e intervenciones indicó un acuerdo que va desde casi perfecto a perfecto, siendo el CCI clasificado como excelente. La aplicación de la prueba de Kruskal-Wallis reveló la ausencia de diferencias estadísticamente significativas entre las evaluaciones.

**Conclusión:**

El estudio validó clínicamente un subconjunto de 53 diagnósticos/resultados y 110 intervenciones de enfermería, demostrando su aplicabilidad en el cuidado de neonatos con catéteres centrales de inserción periférica.

## INTRODUCCIÓN

Actualmente, la incorporación de tecnologías en el ámbito de la atención sanitaria reviste una gran importancia, ya que contribuye a mejorar la calidad de la asistencia y a garantizar una mayor seguridad al paciente, sobre todo en procedimientos invasivos y de elevada complejidad^(1)^. Entre estas tecnologías, destaca el catéter central de inserción periférica (CCIP), un dispositivo indicado para pacientes que necesitan un tratamiento prolongado o que presentan dificultades en el acceso venoso periférico^(2)^.

La eficacia del CCIP se evidencia en la administración de medicamentos y en la nutrición parenteral, lo que lo convierte en una alternativa segura y eficiente. Por lo tanto, garantizar un acceso venoso adecuado es un factor esencial para el éxito del proceso de recuperación clínica del paciente^(1,2)^.

El CCIP está ampliamente indicado para recién nacidos, debido a su facilidad de implantación y a los retos relacionados con la obtención de acceso venoso periférico en este grupo de edad. Su uso se ha vuelto cada vez más frecuente en la asistencia neonatal, especialmente en las unidades de terapia intensiva neonatal (UTIN). Entre sus principales ventajas destacan: la posibilidad de inserción directamente en el entorno de cuidados, sin necesidad de desplazar al paciente; la menor incomodidad para el recién nacido; el menor riesgo de infección en comparación con otros dispositivos de acceso venoso; el prolongado periodo de uso; y la disminución del estrés provocado por múltiples punciones^(2)^.

La inserción del CCIP debe ser realizada por enfermeros debidamente capacitados, según lo establecido en la Resolución n.º 258/2001 del Consejo Federal de Enfermería (COFEN). La responsabilidad del mantenimiento del dispositivo recae en el equipo de enfermería, con el objetivo de reducir posibles complicaciones y garantizar la seguridad del paciente^(3)^.

Es imprescindible tomar diversas precauciones desde el momento de la inserción del CCIP, que requiere el uso adecuado de equipos de protección individual (EPI) y la aplicación rigurosa de la técnica aséptica, hasta las etapas de mantenimiento, que incluyen el cambio de vendajes estériles siempre que sea necesario, la realización de lavados para prevenir la obstrucción del catéter y la desinfección cuidadosa durante la manipulación, con el fin de minimizar el riesgo de infecciones^(1,3)^.

En este contexto, diversas complicaciones pueden dar lugar a la retirada no electiva del catéter, a menudo asociadas al uso de dispositivos venosos. En un ensayo clínico aleatorio realizado con 46 recién nacidos, se observó una tasa de complicaciones que osciló entre el 30,7% y el 62,2%, siendo las más recurrentes: infección (14,2%), obstrucción del catéter (12%), infiltración (4%) y tracción (4%). En un estudio similar, realizado con una muestra de 111 recién nacidos, se observó una incidencia de complicaciones en el 51% de los casos, destacando la colocación inadecuada (25,7%), la flebitis (19,3%) y la oclusión (3,7%) como las más prevalentes^(4)^.

El enfermero desempeña un papel fundamental en la elaboración y ejecución de los cuidados, siendo el Proceso de Enfermería (PE) una herramienta estratégica para promover una atención más organizada, sistematizada y eficiente^(5,6)^. El PE se compone de cinco etapas interdependientes e interrelacionadas: evaluación, diagnóstico, planificación, implementación y evolución de la asistencia de enfermería^(7)^.

Según establece la Resolución n.º 736/2024 del COFEN, es imprescindible que el Proceso de Enfermería esté alineado con los sistemas de estandarización del lenguaje, como la Clasificación Internacional para la Práctica de Enfermería (CIPE®), con el fin de garantizar una mayor consistencia, precisión y calidad en la atención prestada^(7,8)^.

La CIPE^®^ desempeña un papel esencial en la documentación de las acciones del enfermero en la práctica clínica, promoviendo la estandarización, la organización y una mayor precisión en los registros asistenciales. Esta estandarización favorece una comunicación clara y eficaz entre los profesionales de la salud, además de facilitar la sistematización de la asistencia prestada. La CIPE® también permite el desarrollo de Subconjuntos Terminológicos, que reúnen enunciados de diagnósticos/resultados (DE/RE) e intervenciones de enfermería (IE), dirigidos a grupos específicos de pacientes, contribuyendo a la personalización y cualificación de la atención^(9)^.

De este modo, la CIPE® puede definirse como un sistema de comunicación especializado y representativo de la práctica de enfermería, con aplicabilidad tanto en contextos locales como globales, que contribuye directamente a la eficacia del proceso de enfermería. Se trata de un lenguaje estructurado que constituye una estrategia eficiente para la recolección, el almacenamiento y el análisis de datos en enfermería, favoreciendo el fortalecimiento y la valorización de la profesión.

Cabe destacar que, para mejorar la estructuración de los Subconjuntos Terminológicos y ampliar su eficacia, es imprescindible que estos sean validados en el contexto clínico, asegurando su aplicabilidad práctica y relevancia asistencial.

Es importante destacar que, para una estructuración más sólida de los Subconjuntos Terminológicos y para ampliar su eficacia, es fundamental la adopción de un modelo teórico o conceptual que sirva de base para la práctica clínica. Entre los principales referentes teóricos, destaca el modelo de Betty Neuman, que concibe al individuo como un sistema dinámico compuesto por elementos interrelacionados, reconociendo la influencia simultánea de factores internos y externos sobre el estado de salud del paciente^(7,9)^.

En un estudio previo, se llevó a cabo la construcción y validación del contenido de un Subconjunto Terminológico de la CIPE®, dirigido a recién nacidos con CCIP, basado en el modelo teórico de Betty Neuman. La elaboración de este subconjunto surgió de la necesidad de estandarizar los registros de la práctica de enfermería y mejorar la calidad de la documentación asistencial en una UCIN^(7)^.

Posteriormente, se identificó la necesidad de realizar una validación clínica, con el objetivo de garantizar la aplicabilidad y la fiabilidad del subconjunto terminológico en la práctica asistencial. La validación clínica permite verificar si los enunciados desarrollados reflejan adecuadamente las condiciones reales de los recién nacidos con CCIP, garantizando que los DE/RE y las IE sean eficaces en el contexto clínico. Este proceso permite realizar los ajustes pertinentes, contribuyendo a mejorar la precisión de la terminología utilizada y promoviendo una atención más dirigida, segura y cualificada^(10)^.

A pesar de la creciente adopción de la CCIP en la atención neonatal, se observa una carencia significativa en la literatura en cuanto a la validación clínica de subconjuntos terminológicos específicos para esta población. La práctica de la enfermería neonatal requiere instrumentos estandarizados que favorezcan la comunicación eficaz entre los profesionales, además de mejorar la precisión diagnóstica y la implementación de intervenciones dirigidas. Sin embargo, se observa la ausencia de estudios que evalúen la aplicabilidad y la fiabilidad de los enunciados de la CIPE® en recién nacidos en uso de CCIP, lo que dificulta su integración en la práctica clínica y en la documentación sistematizada de la atención.

Ante la brecha identificada en la literatura, esta investigación se propone llenar dicha necesidad mediante la validación clínica de un Subconjunto Terminológico de la CIPE®, dirigido a neonatos ingresados en la UCIN. La propuesta tiene como objetivo ofrecer una base científica que fortalezca la toma de decisiones de los profesionales de enfermería y contribuya a la seguridad y la calidad de la asistencia prestada, justificando así la realización del presente estudio.

Teniendo en cuenta el papel esencial de los estudios de validación, etapa fundamental para evaluar la aplicabilidad de las tecnologías en el contexto de la práctica clínica, es imprescindible garantizar la precisión y la relevancia de los instrumentos utilizados para la toma de decisiones asistenciales. En este sentido, el presente estudio tuvo como objetivo validar clínicamente un subconjunto terminológico de la CIPE^®^ para RN con CCIP, en UTIN.

## MÉTODO

### Diseño y Lugar del Estudio

Se trata de un estudio de validación clínica, en el que se aplicaron estudios de casos realizados de acuerdo con las fases establecidas por el PE. La investigación se llevó a cabo en una UCIN situada en un municipio de la región noreste de Brasil. Además, el estudio siguió las directrices de los Standards for *Quality Improvement Reporting Excellence*(SQUIRE), establecidas por la iniciativa *Enhancing the Quality and Transparency of Health Research* (EQUATOR), que garantizan la calidad y la claridad en la presentación de los resultados^(11)^.

Como base empírica, se utilizó un subconjunto terminológico validado en cuanto al contenido, compuesto por 31 DE/RE y 154 IE, organizados según los principios del modelo teórico de Betty Neuman. La construcción de estos enunciados se basó en la recolección de datos mediante examen físico y en el análisis de los registros de historias clínicas de neonatos en uso de CCIP. Además, el proceso incluyó la verificación de los términos, el mapeo cruzado, la elaboración de los enunciados y la validación del contenido, realizada por un grupo de jueces compuesto por enfermeros especialistas^(7,9)^.

### Población, Criterios de Selección y Definición de la Muestra

La población incluida en la validación clínica consistió en 68 RN en uso de CCIP. Para el cálculo de la muestra, se utilizó la fórmula para poblaciones finitas: [ n = \frac{N \cdot Z2 \cdot (N - 1)) + Z^2 \cdot p \cdot (1 - p)} ] donde «n» representa el tamaño de la muestra, «N» es la población total de recién nacidos hospitalizados con CCIP durante el último año, «Z» corresponde al nivel de confianza establecido (1,96 para un 95% de confianza), «p» se refiere a la proporción poblacional estimada (considerada 0,5 para una mayor variabilidad) y «e» es el margen de error adoptado (10%).

La composición de la muestra siguió criterios de inclusión previamente definidos: RN en uso de CCIP, hospitalizados en UTIN durante el período destinado a la recolección de datos. En cuanto a los criterios de exclusión, se descartaron los neonatos que no utilizaban dicho dispositivo, los transferidos a otras instituciones antes de la finalización de la recolección, así como los RN hijos de madres adolescentes.

La exclusión de los recién nacidos hijos de madres adolescentes se basó en las directrices éticas para la investigación con seres humanos, según lo establecido por la Resolución n.º 466/2012 del Consejo Nacional de Salud (CNS). Dicha normativa considera que los menores de edad, incluidos los adolescentes, no tienen plena capacidad civil, por lo que es necesario un responsable legal para la firma del Término de Consentimiento Libre y Esclarecido (TCLE).

Tras la aplicación de los criterios mencionados, la muestra final estuvo compuesta por 40 recién nacidos. Además de los RN, la muestra también incluyó a cuatro enfermeros especialistas (EE1, EE2, EE3, EE4) en ejercicio en la UTIN, que cumplían los siguientes criterios de inclusión: especialización en neonatología, trabajo en la UTIN, formación específica para la inserción de CCIP y experiencia práctica igual o superior a dos años en la realización de este procedimiento. Los enfermeros ausentes por licencia o vacaciones fueron excluidos de la investigación.

La literatura indica que no existe una definición rígida en cuanto al número de enfermeros especialistas involucrados en la recolección de datos, lo que permite flexibilidad según las necesidades del estudio y los recursos disponibles, y, por esta razón, este número se definió por conveniencia^(12)^.

Los enfermeros especialistas seleccionados para este estudio recibieron una formación estructurada con una duración total de seis horas, impartida por la investigadora principal y distribuida a lo largo de una semana. Esta se llevó a cabo de forma remota y contempló contenidos esenciales para el análisis y la validación clínica de los enunciados del subconjunto terminológico de la CIPE®. Se abordaron temas como la construcción de subconjuntos terminológicos, la aplicación del PE, el juicio clínico y el razonamiento diagnóstico basado en la teoría de Betty Neuman. Además, los participantes recibieron instrucciones detalladas sobre los criterios de evaluación y estandarización de los DE/RE y IE, lo que garantizó la uniformidad en el análisis. Para minimizar los sesgos, la capacitación incluyó ejercicios prácticos y simulaciones de casos clínicos, lo que permitió a los especialistas aplicar los conceptos discutidos en la práctica.

### Recolección de Datos

Para alcanzar el objetivo establecido, el estudio se estructuró y llevó a cabo en tres fases distintas: 1ª) Aplicación del PE a la práctica clínica, que abarca cinco etapas esenciales: evaluación, diagnóstico, planificación, implementación y evolución de la enfermería; 2ª) Estructuración de los estudios de casos, con la decisión clínica de los enunciados de DE/RE e IE; 3ª) Evaluación de la concordancia entre los enfermeros especialistas para garantizar la validez clínica y la precisión de los DE/RE e IE, enumerados a partir de los Estudios de Casos.

El estudio se llevó a cabo entre octubre de 2024 y febrero de 2025. La recolección de datos de los RN se realizó entre octubre y diciembre de 2024, mientras que la etapa de evaluación por parte de los jueces se llevó a cabo entre enero y febrero de 2025.

La recolección de datos se guio por un protocolo, estructurado de acuerdo con las etapas del PE, que estipuló el seguimiento de los participantes desde la inserción del catéter hasta su extracción, con evaluaciones semanales. El equipo responsable, liderado por la investigadora principal, realizó y registró las evaluaciones a los RN, y estructuró los estudios de caso mediante los registros de enfermería.

### Primera Etapa

En la primera fase del estudio, la recopilación de datos y el examen físico fueron realizados por la investigadora, con el apoyo de un instrumento basado en el Modelo de Betty Neuman, lo que garantizó un enfoque estructurado y fundamentado en la teoría. Este instrumento se estructuró en secciones que incluían datos sociodemográficos, historial clínico, diagnósticos, planificación, implementación y evaluación de enfermería. Además, se analizaron exámenes de laboratorio y de imagen para complementar la evaluación clínica.

Inicialmente se realizó una prueba piloto, previamente aprobada por el comité de ética, con el objetivo de verificar la idoneidad del instrumento de recolección. Los participantes en esta prueba no se incluyeron en la muestra final del estudio, garantizando la integridad de los datos recolectados.

Los enunciados de DE/RE se establecieron basándose en indicadores clínicos descritos en las definiciones operativas del subconjunto terminológico de la CIPE®. Para facilitar su organización y accesibilidad, estos enunciados se sistematizaron previamente en un formulario en Google Docs. En los casos en que surgieron situaciones clínicas inéditas, sin un enunciado preexistente, se utilizaron las directrices de la CIPE® versión 2019/2020 para la construcción de nuevos enunciados, garantizando la incorporación de términos de los ejes correspondientes. Además, los DE se determinaron a partir de los aspectos clínicos observados a lo largo del proceso de recolección de datos, lo que proporcionó información para un análisis detallado de la condición de los participantes.

Las etapas de planificación e implementación se llevaron a cabo de acuerdo con los RE establecidos para cada DE, según lo definido por el subconjunto terminológico. Las IE propuestas se orientaron a satisfacer las necesidades identificadas, asegurando un enfoque personalizado y alineado con los objetivos de la atención prestada y las adaptaciones necesarias cuando fuera necesario.

En la etapa de evaluación, se llevó a cabo una nueva recolección de datos y un examen físico, que conformaron la segunda evaluación del estudio. El enfoque de esta fase se centró en la evaluación de los enunciados de DE previamente identificados, lo que permitió verificar la eficacia de las intervenciones y la evolución clínica de los participantes.

### Segunda Etapa

En la segunda fase del estudio, la investigadora desarrolló los estudios de caso y formuló las afirmaciones de DE/RE e IE. Esta información se organizó en una hoja de cálculo de Microsoft Excel®, estructurada en pestañas separadas, cada una de ellas correspondiente a un paciente.

A continuación, las afirmaciones se clasificaron según los niveles de estresores: intrapersonales, interpersonales y extrapersonales.

Tras esta etapa, las hojas de cálculo con los enunciados organizados se enviaron a los cuatro enfermeros especialistas para el proceso de validación clínica.

### Tercera Etapa

En la fase final del estudio, los cuatro enfermeros especialistas realizaron un análisis de concordancia, evaluando la aplicabilidad de los enunciados de DE/RE e IE formulados a partir de los estudios de caso. Cada enfermero recibió una hoja de cálculo correspondiente a un paciente, que contenía todos los enunciados organizados según los niveles de factores estresantes intrapersonales, interpersonales y extrapersonales.

Además de los enunciados, las hojas de cálculo incluían información complementaria, como datos socioeconómicos, historial clínico y observaciones relevantes para el proceso de inferencia diagnóstica. Los enfermeros especialistas evaluaron individualmente la presencia o ausencia de DE/RE e IE en cada hoja de cálculo, asegurando la precisión y validez clínica de los términos.

Durante esta etapa, se consideraron clínicamente validados los DE/RE e IE identificados en los RN en uso de CCIP, según se describen en los estudios de caso, los cuales se incorporaron al subconjunto terminológico. Durante la validación clínica, se añadieron 25 nuevos DE/RE y 22 nuevas IE que contemplaban aspectos fundamentales de la atención neonatal, además de la exclusión de 3 DE/RE y 66 IE del subconjunto inicial por no cumplir los criterios de validación. Como resultado, se obtuvo un subconjunto final compuesto por 53 DE/RE y 110 IE, todos validados con un 100% de concordancia entre los jueces expertos.

El Consejo Internacional de Enfermeras (ICN) anunció una alianza estratégica con la *Systematized Nomenclature of Medicine - Clinical Terms* (SNOMED CT), con el objetivo de integrar la CIPE® a la terminología clínica SNOMED CT.

Como parte de este proceso, se realizó un mapeo cruzado manual entre las dos terminologías, con el fin de actualizar las definiciones del subconjunto terminológico. Para garantizar la precisión conceptual y terminológica, los autores contaron con el apoyo de un traductor jurado en inglés, responsable de alinear los conceptos de la CIPE® con los términos equivalentes en SNOMED CT.

De los 53 DE/RE validados clínicamente al final del estudio, 39 mostraron equivalencia con SNOMED-CT, de los cuales 14 estaban clasificados en el nivel de estresor intrapersonal, 7 en el nivel de estresor interpersonal y 18 en el nivel extrapersonal.

### Análisis y Tratamiento de los Datos

El análisis estadístico del estudio se llevó a cabo con el objetivo de evaluar la concordancia entre los especialistas y verificar posibles diferencias en las evaluaciones de los DE/RE y IE. Para medir la confiabilidad entre evaluadores, se utilizó el coeficiente de Kappa (κ), una medida ampliamente reconocida en estudios de validación clínica, que permite cuantificar la concordancia entre los especialistas más allá de lo que ocurriría por mera casualidad^(13)^.

El Kappa es esencial para garantizar la validez de los enunciados y su aplicabilidad en la práctica clínica, especialmente cuando varios evaluadores analizan los mismos criterios de juicio diagnóstico. Los niveles de concordancia se clasificaron de la siguiente manera: pobre (0 a 0,19), relativa (0,20 a 0,39), moderada (0,40 a 0,59), sustancial (0,60 a 0,79), casi perfecta (0,80 a 0,99) y perfecta (1,00)^(14)^.

Además, para verificar las diferencias estadísticas en las evaluaciones realizadas por los especialistas, se aplicó la prueba de Kruskal-Wallis, un método estadístico no paramétrico adecuado para comparar distribuciones entre tres o más grupos independientes. Esta prueba se eligió por su solidez en situaciones en las que la distribución de los datos puede no cumplir los supuestos de normalidad, lo que garantiza una mayor precisión y fiabilidad en el análisis de los resultados. Así, el uso de estas herramientas estadísticas permitió una evaluación rigurosa de la validez clínica del subconjunto terminológico, asegurando su consistencia y relevancia en la atención neonatal. Se adoptó un nivel de significación de p ≤ 0,05.

Además, se utilizó el coeficiente de correlación intraclase (CCI) para determinar la fiabilidad del subconjunto terminológico en diferentes contextos. La fiabilidad se clasificó como excelente (CCI ≥ 0,75), satisfactoria (CCI entre 0,40 y 0,75) y deficiente (CCI < 0,40)^(13)^.

Los datos relativos a los enunciados de DE/RE se analizaron mediante estadísticas descriptivas simples, expresadas en frecuencias absolutas y relativas. Debido al gran número de enunciados, las IE se agruparon según la categoría del modelo teórico y se discutieron en función de su frecuencia.

Para garantizar la precisión en la organización de los datos, toda la información se registró en una hoja de cálculo, se revisó dos veces antes de exportarla al software Statistical Package for Social Science (SPSS) versión 20.0. Tras la codificación y tabulación, los datos se sometieron a un análisis estadístico y reflexivo, aplicándose la prueba de Kappa mediante la Online Kappa Calculator^(14)^.

En el contexto de esta investigación, se consideraron clínicamente validados los enunciados de DE/RE e IE que cumplían los siguientes criterios: identificación en los pacientes y documentación en los estudios de caso; presencia en el Subconjunto Terminológico^(15)^; coeficiente Kappa clasificado como casi perfecto o perfecto; ausencia de variación significativa en las medias de las evaluaciones realizadas por los especialistas, según la prueba de Kruskal-Wallis; y CCI clasificado como satisfactorio o excelente^(16)^.

### Aspectos Éticos

El presente estudio se llevó a cabo de conformidad con los principios éticos de voluntariedad, anonimato y confidencialidad, de acuerdo con las directrices de la Resolución n.º 466/12 del CNS, que regula la investigación con seres humanos^(17)^. El estudio fue debidamente aprobado por el Comité de Ética en Investigación de una institución federal de educación superior, de conformidad con el dictamen n.º 7.171.001, emitido en el año 2024.

Para garantizar la participación voluntaria e informada en el estudio, todos los participantes, incluidos los responsables de los recién nacidos y los enfermeros especialistas, firmaron el TCLE, asegurando el cumplimiento de las normas éticas y científicas aplicables.

## RESULTADOS

La muestra del estudio estuvo compuesta por 40 RN en uso de CCIP, distribuidos entre ambos sexos, siendo el 65% del sexo femenino y el 35% del sexo masculino. La edad gestacional de los neonatos varió entre 26 y 37 semanas, mientras que el tiempo de hospitalización antes de la inserción del CCIP se produjo entre el primer y el quinto día de hospitalización. En cuanto al peso al nacer, el 65% de los RN presentaban bajo peso, el 15% fueron clasificados como muy bajo peso, el 10% como peso extremadamente bajo y el 10% tenían un peso considerado normal.

Todos los RN incluidos en el estudio presentaban alguna comorbilidad, siendo la disfunción respiratoria la más prevalente, con una incidencia del 75% de los casos, seguida de la prematuridad, observada en el 70% de los participantes. Las principales indicaciones para la inserción del CCIP fueron la antibioterapia, necesaria en el 80% de los casos, la nutrición parenteral, aplicada en el 65% de los recién nacidos, y la administración de fármacos vasoactivos, que representó el 15% de las indicaciones. La estancia media del CCIP fue de 10,23 días, lo que destaca su relevancia en el soporte terapéutico neonatal.

En cuanto al lugar de inserción del CCIP, el 55% de los catéteres se colocaron en los miembros superiores. El procedimiento de inserción requirió una media de 3,72 intentos para obtener resultados satisfactorios. Tras la radiografía para verificar la localización de la punta del catéter, se constató que el 60% se encontraba en posición intracardíaca, lo que hizo necesaria la tracción para su corrección.

En relación con los 31 DE/RE del subconjunto original, se implementaron 28 enunciados (87%), con un promedio de 16 enunciados por estudio de caso, variando entre un mínimo de nueve y un máximo de 28. Tres diagnósticos del subconjunto original, bradicardia, perfusión tisular periférica alterada y riesgo de embolia, no se identificaron en ninguno de los estudios de caso, lo que impidió su validación clínica en el contexto de la atención a neonatos con CCIP.

Cabe destacar que, en los casos en los que surgieron situaciones clínicas inéditas, sin un enunciado preexistente, se utilizaron las directrices de la CIPE® versión 2019/2020 para la construcción de nuevos enunciados, garantizando la incorporación de términos de los ejes correspondientes.

Las Tablas 1, 2 y 3 presentan la distribución de los DE/RE identificados según los niveles de estresores del modelo de Betty Neuman: intrapersonal, interpersonal y extrapersonal. Durante el análisis, los investigadores detectaron la necesidad de incluir 25 DE/RE adicionales, que no estaban contemplados previamente, totalizando al final de la validación clínica 53 DE.

**Tabla 1:** Acuerdo entre enfermeros especialistas en relación a los enunciados del diagnóstico de enfermería del Nivel de Estrés Intrapersonal expresado por el coeficiente Kappa. Natal, RN, Brasil, 2025.

Diagnósticos de Enfermería/Resultados Nivel estresante: intrapersonal	EE 1	EE 2	EE 3	EE 4	Kruskal-Wallis
Riesgo de hemorragia/Control del riesgo de hemorragia	1,00	1,00	0,99	1,00	0,271
Deterioro de la adaptación/Deterioro de la adaptación, Ausente	1,00	1,00	0,97	1,00	0,137
Deterioro de la amplitud de movimiento activo/Rango de movimiento, Efectivo	1,00	1,00	1,00	0,98	0,368
Deterioro de la función del sistema respiratorio/Comp. de la función del sistema respiratorio Comp., Efectiva	1,00	1,00	1,00	1,00	0,092
Deterioro de la actividad psicomotora/Actividad psicomotora, Efectiva	1,00	0,98	0,96	0,96	0,536
Crecimiento retardado/Crecimiento retardado, Ausente	1,00	0,99	0,98	1,00	0,802
Desarrollo deficiente del recién nacido/ Desarrollo del recién nacido, Efectivo	1,00	1,00	0,99	1,00	0,156
Insuficiencia ponderal/Peso dentro de los límites normales	1,00	1,00	1,00	1,00	0,214
Ingesta nutricional deficiente/Ingesta nutricional, Mejorada	0,98	1,00	1,00	1,00	0,406
Riesgo de deterioro de la ingesta nutricional/Ingesta nutricional, Mejorada	1,00	1,00	1,00	1,00	0,529
Deterioro inmunológico/Función del sistema inmunitario, Eficaz	1,00	0,96	1,00	0,98	0,302
Desequilibrio ácido-base/Desequilibrio ácido-base, Ausente	0,98	1,00	0,99	1,00	0,412
Desequilibrio electrolítico/Desequilibrio electrolítico, Ausente	0,99	1,00	1,00	0,98	0,204
Deshidratación/Deshidratación, Ausente	0,98	1,00	1,00	1,00	0,528

**Tabla 2:** Concordancia entre enfermeros especialistas en relación a los enunciados de los diagnósticos de enfermería del Nivel de estresor interpersonal expresado por el coeficiente Kappa. Natal, RN, Brasil, 2025.

Diagnósticos de Enfermería/Resultados Nivel de estresor: interpersonal	EE 1	EE 2	EE 3	EE 4	Kruskal-Wallis
1. Agitación/Agitación, Ausente	0,98	0,94	1,00	0,98	0,332
2. Localización adversa del catéter/Localización del catéter, eficaz	1,00	0,99	1,00	1,00	0,159
3. Lactancia interrumpida/Lactancia, efectiva	0,96	1,00	1,00	0,96	0,538
4. Crianza deficiente/Crianza eficaz	0,98	1,00	1,00	0,98	0,364
5. Proceso familiar comprometido/Proceso familiar, Eficaz	0,99	1,00	1,00	0,99	0,549
6. Proceso familiar interrumpido/Proceso familiar mejorado	1,00	1,00	1,00	0,99	0,320
7. Riesgo de que el vínculo madre/padre e hijo se vea comprometido/Riesgo ausente	1,00	0,98	0,98	1,00	0,134
8. Riesgo de desempeño comprometido de la función parental/Riesgo ausente	1,00	0,96	0,96	1,00	0,450
9. Régimen de medicación complejo/Control	1,00	0,98	0,98	1,00	0,136
10. Riesgo de complicación asociada a la asistencia sanitaria/Control de riesgos	1,00	0,99	0,94	1,00	0,302
11. Riesgo de infección cruzada/Control del riesgo de infección cruzada	1,00	0,99	0,97	1,00	0,142

**Tabla 3:** Concordancia entre enfermeros especialistas en relación a los enunciados de los diagnósticos de enfermería del Nivel Estresante Extrapersonal expresada por el coeficiente Kappa. Natal, RN, Brasil, 2025.

Diagnósticos de Enfermería/Resultados Nivel estresante: extrapersonal	EE 1	EE 2	EE 3	EE 4	Kruskal-Wallis
Dolor agudo/Control del dolor	1,00	0,99	1,00	0,98	0,076
Riesgo de infección/Control del riesgo de infección	1,00	1,00	0,98	1,00	0,052
Infección/Infección, Ausente	1,00	0,96	1,00	1,00	0,073
Hipotermia/Control de la hipotermia	0,98	1,00	1,00	0,98	0,081
Riesgo de alteración de la termorregulación/Control del riesgo	0,98	1,00	0,98	0,98	0,302
Termorregulación, deficiente/Termorregulación, eficaz	0,98	1,00	1,00	0,97	0,232
Edema periférico/Edema periférico, ausente	0,98	1,00	1,00	0,98	0,702
Hematoma/Hematoma, Ausente	1,00	0,99	1,00	0,98	0,304
Integridad de la piel, deficiente/integridad de la piel, eficaz	1,00	1,00	1,00	1,00	0,101
Traumatismo por dispositivo invasivo/Respuesta mejorada al traumatismo por dispositivo	1,00	1,00	1,00	1,00	0,206
Integridad tisular, deteriorada/Integridad tisular, efectiva	0,96	1,00	0,98	1,00	0,449
Riesgo de necrosis/Riesgo de necrosis, ausente	1,00	0,99	1,00	1,00	0,502
Función del Sistema de Regulación, Deteriorada/Función del Sist. de Regulación Regulador, Eficaz	1,00	0,98	0,98	0,98	0,302
Signos vitales, alterados/Signos vitales, en el límite normal	1,00	0,98	1,00	1,00	0,512
Riesgo de deterioro de la función del sistema nervioso/Control de riesgos	0,98	1,00	1,00	0,98	0,074
Riesgo de deterioro de la función del sistema respiratorio/Control del riesgo	1,00	1,00	1,00	1,00	0,205
Riesgo de perfusión tisular, ineficaz/Control del riesgo de perfusión tisular	1,00	0,99	0,98	1,00	0,426
Riesgo de afección neurológica, deteriorado/Control del riesgo	0,99	0,98	0,99	1,00	0,601
Riesgo de Función Cardiovascular, Deteriorada/Control del Riesgo de Función Cardiovascular	0,99	1,00	0,99	1,00	0,083
Riesgo de Ritmo Cardíaco, Deteriorado/Ritmo Cardíaco, Efectivo	0,96	1,00	1,00	1,00	0,092
Alergia al Látex Riesgo/Alergia al Látex, Controlada	0,98	1,00	0,99	1,00	0,193
Alergia/Alergia, Ausente	1,00	0,99	0,98	1,00	0,248
Riesgo de alergia a medicamentos/Riesgo de alergia a medicamentos, controlado	0,97	1,00	1,00	1,00	0,079
Complicación asociada a la asistencia sanitaria/Complication, Absent	1,00	0,98	1,00	0,96	0,082
Obstrucción de catéter/Obstrucción de catéter, Ausente	1,00	1,00	1,00	1,00	0,163
Riesgo de Interacción Adversa con Medicamentos/Risk of Adverse Drug Interaction, Absent	1,00	1,00	1,00	1,00	0,079
Interacción farmacológica, adversa/Interacción farmacológica, adversa, ausente	1,00	1,00	1,00	1,00	0,831
Riesgo de alteración del desarrollo infantil/Control del riesgo	1,00	1,00	1,00	1,00	0,294

En el nivel de estresor intrapersonal, se añadieron DE como: adaptación alterada, amplitud de movimiento activo alterada, función del sistema respiratorio alterada, actividad psicomotora alterada, retraso en el crecimiento, desarrollo alterado del recién nacido, bajo peso, ingesta nutricional alterada, riesgo de ingesta nutricional alterada, compromiso inmunológico, desequilibrio ácido-base y desequilibrio electrolítico.

En cuanto al nivel de estresores interpersonales, se identificaron y añadieron los siguientes DE: ubicación adversa del catéter, lactancia materna interrumpida, paternidad afectada, proceso familiar comprometido, proceso familiar interrumpido, riesgo de compromiso del vínculo madre/padre e hijo, riesgo de compromiso del desempeño del rol parental, régimen farmacológico complejo, riesgo de complicaciones asociadas a la atención sanitaria y riesgo de infección cruzada.

Por último, en el nivel extrapersonal, se añadieron los siguientes DE: riesgo de termorregulación comprometida y riesgo de desarrollo infantil perjudicado, lo que refleja la influencia de los factores ambientales y estructurales sobre la estabilidad del recién nacido.

En relación con el subconjunto original, 66 IE no fueron validadas clínicamente, mientras que 88, equivalentes al 57% del total, fueron aprobadas en la validación clínica. Durante el análisis, se identificó la necesidad de incluir 22 nuevas IE que contemplaran aspectos fundamentales de la atención neonatal, con un total de 110.

Se añadieron 22 IE, a saber: evaluar la edad gestacional, evaluar la integridad de la piel, evaluar el riesgo de apnea, examen físico, evaluar la evolución de la respuesta al manejo del dolor, evaluar la evolución de la respuesta a la termorregulación, evaluar el edema, administrar antibióticos, asesorar sobre la lactancia materna, apoyar la lactancia materna, evaluar la lactancia materna, evaluar el entorno, apoyar a la familia, apoyar el proceso de toma de decisiones familiares, garantizar la continuidad de la atención, evaluar el riesgo de complicaciones adquiridas en el hospital, cuidar el lugar de inserción de dispositivos invasivos, enseñar a la familia sobre la prevención de infecciones, enseñar a la familia sobre la prevención de infecciones cruzadas, enseñar a la familia sobre la susceptibilidad a las infecciones, gestionar el catéter central y mantener el acceso intravenoso.

La inclusión de estas intervenciones refleja la necesidad de enfoques integrales y especializados en la atención a los recién nacidos con CCIP, garantizando la seguridad, la prevención de complicaciones y el apoyo familiar adecuado.

La Tabla 4 presenta el número total de IE identificadas en los participantes de los estudios de caso, así como el porcentaje correspondiente al subconjunto original. El análisis de los DE/RE y las IE evidenció una alta concordancia entre los especialistas, que clasificaron 53 DE/RE y 110 IE como clínicamente válidos.

**Tabla 4:** Intervenciones de enfermería identificadas en las participantes seguidas en los estudios de caso, de acuerdo con los niveles de estresores. Natal, RN, Brasil, 2025.

Nivel de estresor	N	(%)
Intrapersonal	33	82,5%
Interpersonal	37	92,5%
Extrapersonal	40	100%

La prueba estadística de Kruskal-Wallis demostró que no hubo variaciones significativas en las medias de las evaluaciones realizadas por los especialistas, lo que confirma una alineación sustancial entre sus percepciones. Además, el CCI indicó un nivel de fiabilidad excelente (0,98), lo que garantiza la fiabilidad del subconjunto y su aplicabilidad clínica en diferentes contextos de atención neonatal.

## DISCUSIÓN

Los CCIP se introdujeron con el propósito de establecer un acceso venoso central para la administración prolongada de medicamentos y nutrición parenteral^(18)^. Su amplio uso en la práctica clínica como alternativa a los catéteres venosos centrales (CVC) se basa en diversos factores, tales como: menor riesgo de complicaciones intrapara procedimentales, viabilidad económica para su mantenimiento, funcionalidad en contextos ambulatorios y reducción de la incidencia de infecciones del torrente sanguíneo asociadas al catéter^(19)^.

En este contexto, la identificación y el monitoreo de los factores estresantes involucrados en el cuidado del recién nacido en uso de CCIP constituyen estrategias relevantes para la promoción de la salud y la prevención de complicaciones, contribuyendo al fortalecimiento de la seguridad y la calidad de la asistencia prestada.

Así, el subconjunto terminológico propuesto se configura como una herramienta para la identificación y expresión de necesidades de cuidados individualizados. La comunicación clara, las responsabilidades compartidas y la educación continua son componentes críticos de un equipo interprofesional exitoso que atiende a pacientes con CCIP. En consecuencia, al utilizar este instrumento, el enfermero demuestra su competencia profesional a través de registros confiables, que promueven la continuidad y el perfeccionamiento de la asistencia prestada.

Para este propósito, el modelo teórico adoptado desempeñó un papel fundamental en la estructuración del subconjunto, orientándolo desde la perspectiva de la enfermería, con énfasis en la aplicación de los niveles de estresores: intrapersonales, interpersonales y extrapersonales. Cabe destacar que las teorías de enfermería sirven como base fundamental para la creación y evaluación de tecnologías y conocimientos, ya que, por sí solas, representan tecnologías de cuidado indispensables para la implementación de buenas prácticas en enfermería y salud^(6)^.

El análisis de los estresores enfrentados por los RN con CCIP, a la luz de la Teoría de Neuman, reveló la naturaleza multifactorial de las amenazas a la estabilidad sistémica de esta población. Los estresores intrapersonales, interpersonales y extrapersonales demostraron una interconexión significativa, lo que exige enfoques clínicos integrados y sensibles al contexto neonatal.

La validación clínica de los DE/RE e IE basados en esta teoría evidenció una alta concordancia entre los EE, reforzando la pertinencia y aplicabilidad del subconjunto terminológico de la CIPE® en el cuidado del NI con CCIP.

La mayoría de los enunciados se clasificaron como estresores extrapersonales, lo que evidencia el predominio de los factores ambientales y estructurales de la UCIN. A continuación, se destacaron los estresores intrapersonales, relacionados con la vulnerabilidad biológica de los neonatos. Por su parte, los estresores interpersonales presentaron una menor frecuencia, posiblemente debido al enfoque en la atención técnica en este contexto asistencial.

La mayoría de los DE/RE fueron considerados adecuados por los cuatro especialistas, lo que evidencia un sólido consenso sobre su pertinencia para los recién nacidos con CCIP. Términos como riesgo de infección e integridad de la piel alterada fueron validados unánimemente, lo que destaca la preocupación por las complicaciones comunes y la promoción de la atención integral.

Las intervenciones también mostraron un alto grado de concordancia, aunque algunos términos requirieron ajustes semánticos y conceptuales para garantizar la alineación con la CIPE® y el Modelo de Sistemas de Betty Neuman. La validación crítica realizada por expertos reforzó la relevancia clínica, la claridad y la aplicabilidad de los términos en el contexto neonatal.

Una investigación centrada en la construcción y validación de DE destinados a RN prematuros, basada en la CIPE®, reveló un alto nivel de concordancia entre los expertos a lo largo del proceso de validación. De los 146 enunciados formulados, solo uno no alcanzó un índice de validez de contenido (IVC) satisfactorio en relación con la relevancia, por lo que fue excluido de los resultados^(20)^.

En cuanto al nivel de estresores intrapersonales, los enunciados diagnósticos recopilados representan estresores intrapersonales directamente relacionados con las características y condiciones propias del individuo^(21)^. Estos estresores son consecuencia tanto de la inmadurez fisiológica del organismo del recién nacido como de su fragilidad ante las intervenciones necesarias para su supervivencia. Estos factores exigen un enfoque sensible y estrategias de cuidado especializadas para minimizar los impactos negativos en el bienestar infantil.

Los estresores intrapersonales, que se manifiestan internamente en el propio sistema del recién nacido, revelaron una amplia variedad y una fuerte interdependencia, lo que refleja la complejidad de las respuestas individuales frente a las condiciones clínicas y ambientales. Los estudios indican que las complicaciones relacionadas con el uso de la CCIP incluyen riesgo de hemorragias, función inmunológica comprometida, trombosis y desequilibrios metabólicos, como el desequilibrio ácido-base y electrolítico, sobre todo en prematuros extremos (<32 semanas)^(18,22-24)^.

Estas condiciones suelen estar relacionadas con la fragilidad fisiológica, especialmente en aquellos con un peso al nacer inferior a 1500 g o un APGAR bajo, lo que aumenta la susceptibilidad a infecciones y dificultades metabólicas. La tasa de infección del torrente sanguíneo asociada al catéter se registró entre el 10% y el 12% de los casos analizados, lo que evidencia la necesidad de una gestión cuidadosa y una monitorización constante del CCIP. Estos cuidados son esenciales para reducir los riesgos y garantizar la seguridad del paciente durante la terapia intravenosa^(22-24)^.

La atención del enfermero a estos factores estresantes es fundamental para promover una atención integral y dirigida. Al comprender que estas condiciones no son solo consecuencias del entorno o del dispositivo, sino que también están profundamente arraigadas en los aspectos fisiológicos y en el desarrollo del recién nacido, el enfermero puede ajustar sus intervenciones para proporcionar una atención más humanizada y eficaz, alineada con las necesidades individuales del recién nacido. Estas acciones incluyen la monitorización continua, el uso de estrategias no farmacológicas para controlar el dolor y el malestar, el apoyo al desarrollo neuropsicomotor y el ajuste de las terapias nutricionales y fluidoterapéuticas. Además, la identificación temprana y el manejo adecuado de estos factores estresantes garantizan no solo la seguridad, sino también la calidad de la atención, promoviendo el bienestar y el desarrollo saludable del recién nacido.

A nivel de estresores interpersonales, los enunciados de DE/RE reflejaron respuestas específicas de la relación directa que involucra al individuo y al profesional que atiende al paciente^(25)^. En la presente investigación, estos estresores surgieron de las complejas interacciones entre los recién nacidos y los enfermeros. Estos factores pueden ser desencadenantes significativos de estrés, especialmente en contextos de atención sanitaria, donde la relación entre el cliente y los profesionales de la salud puede influir en la estabilidad del sistema.

Durante la inserción del CCIP, la interacción entre el enfermero y el recién nacido puede ser un momento difícil para el RN, generando incomodidad y agitación. Esto se debe a la necesidad de restringir los movimientos del bebé para evitar el desplazamiento del catéter, además del dolor inherente al procedimiento^(26)^. Además, la ubicación incorrecta o la migración del CCIP pueden provocar dolor adicional y dificultar el tratamiento, lo que requiere un monitoreo constante y ajustes en los cuidados. El personal de enfermería debe estar preparado para manejar estas situaciones con sensibilidad para garantizar la comodidad del recién nacido y la confianza de los padres.

En lo que respecta a la interacción entre la madre y el bebé, la interrupción de los procesos familiares, a menudo necesaria debido al uso del CCIP, puede tener efectos adversos en el vínculo entre la madre y el hijo, afectar la confianza de los padres en su capacidad para cuidar al bebé y perjudicar el desarrollo nutricional del recién nacido^(27)^.

Los estudios demuestran que las intervenciones de apoyo a la lactancia materna son fundamentales para minimizar el impacto de esta interrupción. El personal de enfermería desempeña un papel crucial a la hora de ofrecer apoyo emocional e información, lo que contribuye a reducir el estrés de los padres. Además, la gestión eficiente y transparente de los medicamentos, junto con una explicación clara a los padres, puede reducir esta carga de estrés^(28)^.

A nivel de estresores extrapersonales, los enunciados de DE/RE se refieren a influencias externas al paciente que pueden afectar su estabilidad y bienestar^(25)^. Estos estresores están asociados a elementos ambientales y circunstanciales, como políticas, sistemas de salud, condiciones socioeconómicas y recursos disponibles. Actúan indirectamente sobre el sistema del recién nacido y pueden influir en su capacidad de adaptación a otros estresores.

Las afirmaciones diagnósticas extrapersonales, como las complicaciones asociadas a la atención sanitaria, los regímenes farmacológicos complejos o las interacciones farmacológicas adversas, requieren una vigilancia constante para garantizar que el tratamiento se administra correctamente. La complejidad del tratamiento, sumada a la administración de numerosos medicamentos durante el uso de la CCP, también representa un factor de estrés para el recién nacido, lo que afecta su respuesta fisiológica y aumenta la probabilidad de complicaciones^(29)^.

Basándose en las evaluaciones clínicas realizadas, la aplicación sistemática de los cuidados, ejemplificada por el uso del subconjunto, respalda el juicio clínico del enfermero y permite una planificación alineada con las particularidades de cada paciente.

Ante la temática propuesta, el enfermero, en su actuación profesional, lleva a cabo el juicio clínico basándose en el análisis de las respuestas del paciente, empleando DE para dirigir y calificar el proceso de cuidado. El empleo de lenguajes estandarizados, como el CIPE®, permite describir este conocimiento y aplicar IE específicas de acuerdo con las necesidades del paciente^(13,30,31)^. Dichas intervenciones se centran en la formación continua del equipo, la educación y los cambios de comportamiento, lo que contribuye al éxito durante toda la terapia intravenosa del recién nacido que implica el uso del CCIP como dispositivo de elección^(32-34)^.

El impacto del subconjunto discutido en la práctica asistencial está vinculado a su relevancia como referencia para los enfermeros que atienden a neonatos en uso de CCIP en la UCIN. Este instrumento se destaca como una guía eficaz para la elaboración de planes de cuidados individualizados. Las instituciones de salud pueden adoptar subconjuntos como estrategia para elevar la calidad de la asistencia, empleando los enunciados como referencia e integrando evidencias para fundamentar la toma de decisiones clínicas, mejorar la asistencia, utilizando los enunciados como indicativos e incorporando evidencias obtenidas que pueden convertirse en indicadores clínicos de calidad^(35)^.

Cabe destacar que la evaluación realizada por los profesionales al utilizar el subconjunto construido resultó de gran relevancia. En este contexto, se observó una mayor homogeneidad en las evaluaciones, lo que sugiere una mayor eficiencia en el análisis de los pacientes. Esto fue posible gracias a la base científica proporcionada, que validó los indicadores utilizados para medir resultados concretos, lo que sirvió de apoyo para la elaboración del plan de cuidados de enfermería.

En cuanto a las limitaciones del estudio, la selección de una población específica puede haber reducido el alcance de los hallazgos, lo que dificulta su generalización. Además, el hecho de que el estudio se haya restringido a una sola región y contexto introdujo una característica cultural singular, que debe considerarse cuidadosamente al analizar los resultados, garantizando una comprensión más precisa de los hallazgos del estudio.

La implementación del subconjunto terminológico validado para neonatos con CCIP en la UCIN requiere un enfoque estructurado e integrado en la rutina de los profesionales de enfermería. En primer lugar, es fundamental promover la formación continua para capacitar a los enfermeros en el uso correcto de los enunciados de DE/RE e IE de la CIPE®. La realización de talleres y simulaciones clínicas puede ayudar a consolidar el razonamiento clínico necesario para identificar los indicadores específicos del diagnóstico de localización adversa de la CCIP y tomar decisiones acertadas.

Además de la capacitación, la integración del subconjunto en los sistemas electrónicos de historias clínicas puede facilitar el acceso de los profesionales a las terminologías estandarizadas, garantizando una mayor precisión en la documentación de la atención. El uso de software de apoyo a la toma de decisiones clínicas, con sugerencias automatizadas de diagnósticos e intervenciones basadas en los hallazgos clínicos, puede optimizar la práctica y minimizar las inconsistencias en los registros de enfermería. Esta integración tecnológica también favorece la comunicación interprofesional, al permitir que diferentes miembros del equipo de salud accedan a información estandarizada y actualizada sobre la atención prestada al paciente.

Otro aspecto esencial para la implementación exitosa del subconjunto terminológico es la creación de protocolos de atención basados en definiciones validadas. La incorporación de estos términos en los manuales de conducta de la UCIN puede garantizar una mayor uniformidad en las prácticas de enfermería y reducir las variaciones en el enfoque de los casos clínicos. Además, los criterios de validación pueden servir de base para auditorías internas, lo que permite supervisar el cumplimiento de las mejores prácticas e identificar oportunidades de mejora en la atención prestada.

Por último, la realización de estudios multicéntricos puede ser un paso estratégico para ampliar la aplicabilidad del subconjunto validado en diferentes instituciones de salud. La replicación del modelo asistencial en otras UCIN permitirá evaluar su eficacia en diversos contextos, reforzando su utilidad en la práctica clínica. Para garantizar la continuidad de la actualización, se recomienda que los profesionales involucrados en la investigación participen en redes de colaboración científica, donde puedan compartir hallazgos y contribuir a la evolución del conjunto terminológico de la CIPE®.

La implementación eficaz de este subconjunto representa un avance significativo en la estandarización del lenguaje especializado de la enfermería neonatal, fortaleciendo la toma de decisiones de los profesionales, mejorando la seguridad del paciente y garantizando una atención más calificada a los recién nacidos en uso de CCIP.

## CONCLUSIÓN

La investigación evidenció la validación clínica de un subconjunto compuesto por 53 DE/RE y 110 IE de la CIPE®, dirigido al cuidado de RN en uso de CCIP en la UTIN. Este subconjunto es el resultado de la validación clínica del 90,3% de los 31 DE/RE y del 57% de los 154 IE originalmente presentes en el subconjunto terminológico inicial, además de la inclusión de 25 nuevos DE/RE y 22 nuevos IE y la exclusión de 3 DE/RE y 66 IE.

Los enunciados mostraron índices de concordancia clasificados como casi perfectos y perfectos, además de una concordancia global excelente, sin evidencia de diferencias estadísticamente significativas entre las evaluaciones realizadas por los especialistas. De este modo, la elaboración de estos enunciados empíricos, junto con el proceso de validación clínica, contribuye a la adecuación de los enunciados de enfermería a poblaciones específicas, proporcionando un medio eficaz para la evaluación mejorada de las intervenciones de enfermería.

A la luz de los resultados de este estudio, futuras investigaciones podrían explorar el impacto del subconjunto terminológico en la formación de los profesionales, investigando cómo su uso puede mejorar el juicio clínico de los enfermeros y contribuir a la estandarización de la atención neonatal. Otra dirección prometedora es el análisis de la mejora en la comunicación interprofesional, verificando cómo la adopción de un lenguaje estandarizado puede optimizar el intercambio de información entre enfermeros, médicos y otros profesionales involucrados en la atención neonatal, potenciando la integración de los equipos y la calidad de la atención prestada. Estas investigaciones futuras podrían fortalecer aún más la aplicabilidad del subconjunto terminológico, consolidando su relevancia en la enfermería neonatal y ampliando su impacto en la práctica clínica.

## DISPONIBILIDAD DE DATOS

Los datos utilizados durante este estudio están disponibles del autor correspondiente previa solicitud razonable.
